# Magnetic field effects in biology from the perspective of the radical pair mechanism

**DOI:** 10.1098/rsif.2022.0325

**Published:** 2022-08-03

**Authors:** Hadi Zadeh-Haghighi, Christoph Simon

**Affiliations:** ^1^ Department of Physics and Astronomy, University of Calgary, Calgary, Alberta, Canada T2N 1N4; ^2^ Institute for Quantum Science and Technology, University of Calgary, Calgary, Alberta, Canada T2N 1N4; ^3^ Hotchkiss Brain Institute, University of Calgary, Calgary, Alberta, Canada T2N 1N4

**Keywords:** magnetic field effects in biology, isotope effects in biology, reactive oxygen species, radical pair mechanism, quantum biology, spin chemistry

## Abstract

Hundreds of studies have found that weak magnetic fields can significantly influence various biological systems. However, the underlying mechanisms behind these phenomena remain elusive. Remarkably, the magnetic energies implicated in these effects are much smaller than thermal energies. Here, we review these observations, and we suggest an explanation based on the radical pair mechanism, which involves the quantum dynamics of the electron and nuclear spins of transient radical molecules. While the radical pair mechanism has been studied in detail in the context of avian magnetoreception, the studies reviewed here show that magnetosensitivity is widespread throughout biology. We review magnetic field effects on various physiological functions, discussing static, hypomagnetic and oscillating magnetic fields, as well as isotope effects. We then review the radical pair mechanism as a potential unifying model for the described magnetic field effects, and we discuss plausible candidate molecules for the radical pairs. We review recent studies proposing that the radical pair mechanism provides explanations for isotope effects in xenon anaesthesia and lithium treatment of hyperactivity, magnetic field effects on the circadian clock, and hypomagnetic field effects on neurogenesis and microtubule assembly. We conclude by discussing future lines of investigation in this exciting new area of quantum biology.

## 1. Introduction

Sensitivity to weak magnetic fields is abundant throughout biology, as discussed in numerous review articles [1–24]. Effects of either static or oscillating weak magnetic fields have been reported on the circadian clock, electron transfer in cryptochrome, stem cells, calcium concentration, the brain’s functions such as action potentials, reactive oxygen species (ROS), development, neuronal activities, DNA, memory, anxiety, analgaesia, genetics and many other functions (see §2). Despite the wealth of observations, thus far, there is no clear explanation for the mechanism behind these phenomena. This is mainly due to the fact that the corresponding energies for such effects are far smaller than thermal energies.

However, there is a promising quantum physics (or spin chemistry) concept that can account for the effects of such weak fields, namely the radical pair mechanism [25,26]. This mechanism, which is an example of the emerging field of quantum biology [27–31], has been studied in significant detail in the comparatively narrow context of bird magnetoreception [32–39], where it is accepted as one of the leading potential explanations for how birds sense magnetic fields, and in particular the Earth’s magnetic field, for the purpose of navigation. It is known that birds and amphibians, and in all likelihood other vertebrates, have not one but two magnetoreception mechanisms, a magnetite-based detector that provides the high sensitivity necessary for sensing weak spatial gradients in the magnetic field [40,41] and a light-dependent magnetic compass that underlies a magnetic map sense [42]. The latter is thought to be based on the radical pair mechanism [43,44].

The radical pair mechanism involves magnetically sensitive intermediate molecules, so-called radical pairs [25,43,45–49]. The key ingredient is the spin correlation between two unpaired electrons, one on the donor molecule and the other on the acceptor molecule. Depending on the initial spin configuration of the donor and acceptor molecules, this initial spin correlation of the radical pair will be either a singlet (S) or a triplet (T) state, which are, respectively, spin-0 and spin-1 states (see §3.1 for further discussion). Due to the spin interactions with its environment (in particular with external magnetic fields and with nearby nuclear spins), the state of the radical pair will oscillate between S and T states [26,50]. Each spin state, S and T, can lead to different reaction products, providing an example of spin chemistry [51,52]. The energies induced by the above-mentioned magnetic fields are hundreds of thousands of times smaller than thermal energies, *k*_*B*_*T* (*k*_*B*_ is Boltzmann constant and *T* is temperature), which are associated with motions, rotation and vibrations in biological environments. In thermal equilibrium, the energies required to alter the rate or yield of a chemical transformation should be at least comparable to *k*_*B*_*T*. Due to this, the radical pair mechanism was originally ignored in the context of physiology. However, the situation differs in systems far from thermal equilibrium, which is the case for radical pairs [43]. Sensitivity to weak magnetic fields is one of the key properties of radical pair reactions. Nowadays, many research laboratories study the role of radical pairs in (bio)chemical reactions [26,52–56].

Recent studies have proposed roles for radical pairs beyond avian magnetoreception, in particular in xenon-induced anaesthesia [57], lithium effects on mania [58], magnetic field and lithium effects on the circadian clock [59], and hypomagnetic field effects on microtubule reorganization [60] and neurogenesis [61] (where hypomagnetic fields are fields much weaker than that of the Earth). Here, we suggest that the radical pair mechanism is in fact quite common in biology, and that it may provide an explanation for many of the weak magnetic field effects on physiological functions that have been observed.

This paper, which is part review and part perspective article, is organized as follows. Section 2 briefly surveys studies reporting effects of low-intensity magnetic fields on biological systems, including effects of static (§2.1), hypomagnetic (§2.2) and oscillating (§2.3) magnetic fields. We further survey studies on isotope effects in biology from a spin perspective. In §3, we discuss how the radical pair mechanism can account for static, hypomagnetic and oscillating magnetic field effects. Section 3.4 reviews possible candidate molecules for radical pair formation in biological systems. In §4, we review the above-mentioned recent studies on the possible biological roles of radical pairs beyond avian magnetoreception. Section 5 discusses important directions for further investigation.

## 2. Magnetosensitivity in biology

There is a considerable amount of research investigating magnetic field effects on biological functions [22,62–70]. In the following, we review the effects of low-intensity magnetic fields on biology. We organize this section based on the type of magnetic fields, namely static magnetic fields, hypomagnetic fields and oscillating magnetic fields. Isotope effects in biology, which can be related to nuclear magnetic moments, are also discussed at the end of this section.

### 2.1. Static magnetic field

#### 2.1.1. Cryptochrome

In the context of avian magnetoreception in animals, the canonical proteins are cryptochromes [43,48]. Maeda *et al.* demonstrated that photo-induced flavin–tryptophan radical pairs in cryptochrome are magnetically sensitive [71]. Moreover, Ahmad *et al.* observed that hypocotyl growth inhibition in higher plants are sensitive to the magnetic field, where such responses are linked to cryptochrome-dependent signalling pathways [72]. Sheppard *et al.* reported that magnetic fields of a few millitesla could influence photo-induced electron transfer reactions in *Drosophila* cryptochrome [73]. Further, Marley *et al.* showed that a static magnetic field of 100 mT substantially affected seizure response in *Drosophila* larvae in a cryptochrome-dependent manner [74]. In addition, using a transgenic approach, Foley *et al.* showed that human cryptochrome-2 has the molecular capability to function as a light-sensitive magnetosensor [75]. Applying a 0.5 mT magnetic field, Ahmad and co-workers reported that cryptochrome responses were enhanced by the magnetic field, including dark-state processes following the cryptochrome photoreduction step [76,77]. Further, there have been extensive studies on the radical pair mechanism for cryptochrome(s) [43,47]. Table 1 summarizes static magnetic field effects on various biological functions.

**Table 1.** Static magnetic field effects on different biological functions.

system	magnetic field	references
**cryptochrome**		
cryptochrome responses enhanced	0.5 mT	Pooam *et al.* [76]
cryptochrome responses enhanced	0.5 mT	Hammad *et al.* [77]
seizure response in *Drosophila* (cryptochrome-dependent)	further, 100 mT	Marley *et al.* [74]
photo-induced electron transfer reactions in *Drosophila* cryptochrome	a few mT	Sheppard *et al.* [73]
body size increase and in *Drosophila melanogaster*	0.4–0.7 mT	Giorgi *et al.* [78]
decrease in wing size in *Drosophila melanogaster*	35 mT	Stamenkovi-Radak *et al.* [79]
**circadian clock**		
circadian clock in *Drosophila melanogaster*	<0.5 mT	Yoshii *et al.* [80]
**stem cell**		
stem cell-mediated growth	<1 mT	Huizen *et al.* [81]
proliferation/migration/differentiation in human dental pulp stem cells	1/2/4 mT	Zheng *et al.* [82]
bone stem cells *in vitro*	0.5–30 mT	Abdolmaleki *et al.* [83–85]
**calcium**		
Ca^2+^ influx	0.6 mT	Fanelli *et al.* [86]
myosin phosphorylation in a cell-free preparation (Ca^2+^-dependent)	0.2 mT	Markov & Pilla [87]
Ca^2+^ concentration/morphology in cell lines	6 mT	Tenuzzo *et al.* [88]
Ca^2+^ concentration in *in vitro* aged human lymphocytes	6 mT	Tenuzzo *et al.* [89]
cell shape, cell surface, sugar residues, cytoskeleton and apoptosis	6 mT	Chionna *et al.* [90]
**neurons and brain**		
blocked sensory neuron action potentials in the somata of adult mouse	10 mT	McLean *et al.* [91]
symptomatic diabetic neuropathy	50 mT	Weintraub *et al.* [92]
**ROS**		
increased intercellular ROS in human neuroblastoma cells	2.2 mT	Calabro *et al.* [93]
increased intercellular ROS in human neuroblastoma cells	31.7–232 mT	Vergallo *et al.* [94]
increased H_2_O_2_ level in embryoid bodies	1–10 mT	Bekhite *et al.* [95]
ROS increase in mouse cardiac progenitor cells	0.2–5 mT	Bekhite *et al.* [96]
elevated H_2_O_2_ in diploid embryonic lung fibroblast cell	230–250 mT	Sullivan *et al.* [97]
increase of H_2_O_2_ in the human fibrosarcoma cancer cell	45−60 μT	Martino& Castello [98]
increased H_2_O_2_ production of human peripheral blood neutrophils	60 mT	Poniedzialek *et al.* [99]
ROS levels in cancer cells	10 mT	Verdon [100]
type 2 diabetes via regulating cellular ROS	3 mT	Carter *et al.* [101,102]
ROS changes in stem cell-mediated growth	<1 mT	Huizen *et al.* [81]
mitochondrial electron transport chain activity	0–1.93 mT	Sheu *et al.* [103]
**others**		
flavin adenine dinucleotide photochemistry	<20 mT	Antill *et al.* [104]
enzymatic ATP production	80 mT	Buchachenko *et al.* [105]
chlorophyll fluorescence/nutrient content of *Hordeum vulgare* L.	20/42/125/250 mT	Ercan *et al.* [106]
antioxidant defense system of plant cells	10/30 mT	Sahebjamei *et al.* [107]
enhance the killing effect of adriamycin on K562 cells.	8.8 mT	Hao *et al.* [108]
regeneration and plant growth of shoot tips	2.9–4.6 mT	Atak *et al.* [109]
accelerated loss of integrity of plasma membrane during apoptosis	6 mT	Teodori *et al.* [110]
macrophagic differentiation in human pro-monocytic U937 cells	6 mT	Pagliara *et al.* [111]
cell proliferation and cell death balance	0.5 mT	Buemi *et al.* [112]
growth and sporulation of phytopathogenic microscopic fungi	1 mT	Nagy *et al.* [113]

#### 2.1.2. Genetics

It is known that exposure to magnetic fields has genetic consequences [114]. Giorgi *et al.* showed that chronic exposure to magnetic fields (0.4–0.7 mT) increased the body size and induced lethal mutations in populations of *Drosophila melanogaster* [78]. Furthermore, a magnetic field of 35 mT decreased the wing size in *Drosophila melanogaster* [79] (table 1).

#### 2.1.3. Circadian clock

It has been shown that magnetic fields can modulate the circadian clock [115–117]. Yoshii *et al.* [80] showed that the effects of static magnetic fields affected the circadian clock of *Drosophila* and reported that exposure to these fields slowed down the clock rhythms in the presence of blue light, with a maximal change at 300 μT, and reduced effects at both lower and slightly higher field strengths. We discuss this observation further from the perspective of the radical pair mechanism in §4.3 (table 1).

#### 2.1.4. Stem cells

Static magnetic fields have been commonly used in medicine as a tool to increase wound healing, bone regeneration and as a component of magnetic resonance techniques. However, recent data have shed light on deeper mechanisms of static magnetic field action on physiological properties of different cell populations, including stem cells. It is known that static magnetic fields can increase wound healing and bone regeneration [8]. Huizen *et al.* reported that weak magnetic fields (less than 1 mT) alter stem cell-mediated growth, where changes in ROS were implicated [81]. The authors suggested that the radical pair mechanism may be the potential explanation for their observations. Zheng *et al.* showed that a static magnetic field of 1, 2 or 4 mT regulated proliferation, migration, and differentiation of human dental pulp stem cells [82]. It is also known that applied static magnetic fields (0.5–30 mT) affect stem cells *in vitro* [83–85] (table 1).

#### 2.1.5. Calcium

Fanelli *et al.* reported that magnetic fields allow the indefinite survival and replication of the cells hit by apoptogenic agents. The anti-apoptosis effect was found to be mediated by the ability of the fields to increase Ca^2+^ influx from the extracellular medium. In that experiment, the geomagnetic field was not shielded. They found 0.6 mT to be the minimal intensity required to detect an anti-apoptotic effect [86]. Moreover, it has been shown that weak static magnetic fields can influence myosin phosphorylation in a cell-free preparation in a Ca^2+^-dependent manner [87]. Tenuzzo and colleagues observed that exposure to a 6 mT static magnetic field influenced Ca^2+^ concentration and bcl-2, bax, p53 and hsp70 expression in freshly isolated and *in vitro* aged human lymphocytes [89]. Further, Chionna *et al.* showed that exposure to a static magnetic field of 6 mT of Hep G2 cells resulted in time-dependent modifications in cell shape, cell surface, sugar residues, cytoskeleton and apoptosis [90]. They reported that after 24 h exposure, the cells had a less flat shape due to partial detachment from the culture dishes. They further observed that microfilaments and microtubules were modified in a time-dependent manner. They also suggested that the induced apoptosis was likely due to the increment of Ca^2+^ during exposure. In another study, Tenuzzo and co-workers showed that cell viability, proliferation, intracellular Ca^2+^ concentration and morphology in several primary cultures and cell lines can be influenced by a 6 mT magnetic field [88] (table 1).

#### 2.1.6. Neurons and brain

Exposure to static magnetic fields can have impacts on various brain functions. McLean *et al.* reported that a static magnetic field in the 10 mT range blocked sensory neuron action potentials in the somata of adult mouse dorsal root ganglion neurons in monolayer dissociated cell culture [91]. It has also been shown that exposure to a transcranial static magnetic field over the supplementary motor area can modulate resting-state activity and motor behaviour associated with modulation of both local and distant functionally connected cortical circuits [118]. Static magnetic field exposure can also affect the production of melatonin [119–122], the pineal gland [123,124], and cause functional alterations in immature cultured rat hippocampal neurons [125]. Further, Dileone *et al.* observed that an applied transcranial static magnetic field can induce dopamine-dependent changes of cortical excitability in patients with Parkinson’s disease [126]. In addition, neuron firing frequency can also be affected by static magnetic field intensity [127,128]. There exist a considerable number of studies indicating the effects of applied magnetic field on pain sensitivity (nociception) and pain inhibition (analgesia) [129]. Additionally, it has been known that a static magnetic field (50 mT) can influence symptomatic diabetic neuropathy [92] (table 1).

#### 2.1.7. Reactive oxygen species

ROS are the collection of derivatives of molecular oxygen that occur in biology, which can be categorized into two types, free radicals and non-radical species. The non-radical species are hydrogen peroxide (H_2_O_2_), organic hydroperoxides (ROOH), singlet molecular oxygen (^1^O_2_), electronically excited carbonyl, ozone (O_3_), hypochlorous acid (HOCl, and hypobromous acid HOBr). Free radical species are superoxide anion radical (O_2_^•−^), hydroxyl radical (^•^OH), peroxyl radical (ROO^•^) and alkoxyl radical (RO^•^) [130]. Any imbalance of ROS can lead to adverse effects. H_2_O_2_ and O_2_^•−^ are the main redox signalling agents. It is now well known that ROS are essential for physiology as functional signalling entities. H_2_O_2_ plays a crucial role in redox regulation of biological functions, where its intracellular concentration is under tight control. The cellular concentration of H_2_O_2_ is about 10^−8^ M, which is almost a thousand times more than that of O_2_^•−^. Transmembrane NADPH oxidases (NOXs) [131,132] and the mitochondrial electron transport chain (ETC) [133,134] are the major sources of O_2_^·−^ and H_2_O_2_.

In a considerable number of studies, magnetic field effects in biology are accompanied with oxidative stress [15,135,136], which is *an imbalance between oxidants and antioxidants in favour of the oxidants, leading to a disruption of redox signalling and control and/or molecular damage.* [137–139]. Studies found that exposure to static magnetic fields of 2.2 mT [93] and 31.7–232 mT [94] increased the intercellular ROS in human neuroblastoma cells. Furthermore, De Nicola *et al.* observed that the intracellular ROS level in human monocyte tumour cells was raised when exposed to a static magnetic field [140]. Further, Bekhite *et al.* reported that static magnetic field exposure (1–10 mT) increased the H_2_O_2_ level in embryoid bodies [95]. Later, the same group found an induced increase of ROS in cardiac progenitor cells derived from mouse cells by a 0.2–5 mT static magnetic field, where ROS was suggested to be generated by NADPH oxidase [96]. Sullivan *et al.* reported that 230–250 mT of a magnetic field elevated H_2_O_2_ in diploid embryonic lung fibroblast cell [97]. Upon exposure to 45–60 μT, Martino and Castello observed an increase of H_2_O_2_ in the human fibrosarcoma cancer cell, which can be suppressed by reducing the geomagnetic field’s strength [98]. Further studies show that exposure to a 60 mT magnetic field increased H_2_O_2_ production of human peripheral blood neutrophils [99]. It has also been reported that the effects of an applied magnetic field of 10 mT on DOXO-induced toxicity and proliferation rate of cancer cells are correlated to ROS levels [100]. Furthermore, Carter *et al.* observed that a 3 mT static magnetic field can influence type 2 diabetes via regulating cellular ROS [101,102]. Pooam *et al.* showed that applying a low intensity static magnetic field modulated ROS generation in HEK293 cells. The authors suggested that the radical pair mechanism may explain that observation [141]. In a recent work, Sheu and co-workers reported that static low intensity magnetic fields can regulate mitochondrial ETC activity and thus enhance mitochondrial respiration [103]. They observed that exposure to magnetic fields of 0–1.93 mT of mitochondria isolated from adult rat hearts produced a bell-shape increase in the respiratory control ratio with a maximum at 0.50 mT and a return to baseline at 1.50 mT. It was further observed that the magnetic field affected only the activity of the complexes 2, 3 and 5 but not 1 of the mitochondrial ETC and several enzymes of the tricarboxylic acid cycle. The authors suggested that the low intensity magnetic field effects on the mitochondrial respiratory activity may be explained by the radical pair mechanism. Huizen and co-workers showed that weak magnetic fields (less than 1 mT) changed stem cell-mediated growth, where changes in ROS were implicated [81].

#### 2.1.8. Others

Ikeya *et al.* reported that exposure to magnetic fields influenced autofluorescence in cells involving flavins [142]. Studies also showed that static magnetic fields can affect the photoactivation reaction of *E. coli* DNA photolyase [143]. Moreover, Giachello *et al.* observed that applying static magnetic fields on blue light activated cryptochromes in *Drosophila* neurons resulted in an elevation of action potential firing [144]. Further, it is also known that the chemiluminescence intensity in Madin–Darby canine kidney cells is magnetic field dependent [145], where ROS are implicated. In solutions, flavin adenine dinucleotide is the key cofactor of cryptochrome. Antill and co-workers showed that flavin adenine dinucleotide photochemistry in solution is magnetic field sensitive (less than 20 mT) even at physiological pH and higher [104].

Buchachenko *et al.* reported that applying 80 mT static magnetic field affected enzymatic ATP production [105]. Recently, Ercan *et al.* showed that exposure to magnetic fields (20, 42, 125 and 250 mT) can affect the magnetic properties, germination, chlorophyll fluorescence and nutrient content of barley (*Hordeum vulgare* L.) [106]. Further, it is observed that exposure to magnetic fields (10 and 30 mT) can deteriorate the antioxidant defence system of plant cells [107]. Hao *et al.* reported that exposure to an 8.8 mT static magnetic field can enhance the killing effect of adriamycin on K562 cells [108]. It is also observed that exposure to magnetic fields (2.9–4.6 mT) of soya bean tissue culture enhances the regeneration and plant growth of shoot tips [109]. Teodori *et al.* showed that exposure of HL-60 cells to a 6 mT static magnetic field accelerated loss of integrity of plasma membrane during apoptosis [110]. It has been shown that exposure of human pro-monocytic U937 cells to a static magnetic field (6 mT) decreased the degree of macrophagic differentiation [111]. Buemi *et al.* report that exposure to a 0.5 mT magnetic field of renal cell cultures and cortical astrocyte cultures from rats influenced cell proliferation and cell death balance [112]. They concluded that such magnetic field effects were cell type-dependent. It has been shown that exposure to magnetic fields (1 mT) significantly affected growth and sporulation of phytopathogenic microscopic fungi [113].

Surma *et al.* found that the application of a weak static magnetic field with intensities only a few times that of the geomagnetic field can accelerate the development of skeletal muscle cells, resulting in the formation of multinuclear hypertrophied myotubes [146]. They further reported that these effects were accompanied by a 1.5- to 3.5-fold rise in the concentration of intracellular [Ca^2+^]_*i*_.

### 2.2. Hypomagnetic field

Earth’s geomagnetic field, ranging from approximately 24 to 66 μT depending on latitude [147], can have critical roles in numerous biological processes. Shielding the geomagnetic field, called hypomagnetic field, is known to cause biological effects [19,21,23,148–152].

It has also been suggested that the apparent cycle of mass extinction on Earth [153] may be related to the geomagnetic field fluctuation [154]. Decades ago, the first studies on the effects of hypomagnetic field on humans were conducted, motivated by the concerns around the health of astronauts in outer space [155–158]. These studies concluded that exposure to hypomagnetic fields had adverse effects on human health. Besides hypomagnetic field effects on animal and human cells and tissues, deprivation in geomagnetic field can influence the development of plants as well [151,152]. The geomagnetic field seems to play essential roles in living organisms, and diminishing or removing it could result in adverse consequences.

It was shown that exposure to hypomagnetic fields decreased the size and number of *Staphylococcus aureus* [159]. Exposure to hypomagnetic fields can also influence early developmental processes of newts (*Cynops pyrrhogaster*) [160], early embryogenesis [161,162], development of *Xenopus* [163], cryptochrome-related hypocotyl growth and flowering of *Arabidopsis* [164,165], development and reproduction of brown planthopper [166], mortality [167] and anhydrobiotic abilities [168] in tardigrades.

It was observed that the circadian clock in fiddler crabs and other organisms [169], including human [170] and birds [171] can be influenced by exposure to hypomagnetic fields.

Zhang *et al.* showed that long-term exposure to hypomagnetic fields adversely influenced adult hippocampal neurogenesis in mice [172]. They further observed that these effects were accompanied by reductions in ROS levels. Moreover, Wang *et al.* observed that exposure to hypomagnetic fields (10–100 nT) caused disorders in tubulin self-assembly [173]. They show that the absorbance for monitoring tubulin self-assembly was altered by exposure to hypomagnetic fields. We discuss both these observations from the perspective of the radical pair mechanism in the following (see §§4.4 and 4.5). Furthermore, Baek *et al.* reported that exposure to hypomagnetic fields influenced DNA methylation *in vitro* in mouse embryonic stem cell (ESC) culture [174]. Upon exposure to a hypomagnetic field ESC morphology remained undifferentiated while under exposure to the geomagnetic field, ESCs exhibited differentiation. Moreover, Ikenaga and co-workers reported that genetic mutation in *Drosophila* during space flight [175]. Further, Martino and co-workers reported that reducing the geomagnetic field to 6–13 μT resulted in significantly altered cell cycle rates for multiple cancer-derived cell lines [176]. Belyavskaya observed that hypomagnetic conditions included reduction of the meristem, disruption of protein synthesis and accumulation of lipids, reduction in organelle growth, the amount of phytoferritin in plastids and crista in mitochondria [177]. Further, the effects of zero magnetic field on human VH-10 fibroblasts and lymphocytes were observed by Belyaev *et al.* [178]. They concluded that exposure to hypomagnetic fields caused hypercondensation and decondensation of chromatin. Studies conducted by NASA revealed that exposure to hypomagnetic fields decreased enzyme activity in cells obtained from mice [179].

Yan *et al.* show that reducing the magnetic field to less than 0.5 μT significantly lengthened larval and pupal development durations, increased male longevity, and reduced pupal weight, female reproduction, and the relative expression level of the vitellogenin gene in *Mythimna separata* [180]. In addition, they observed that exposure to the hypomagnetic field had adverse effects on the mating ratio of *M. separata* adults. They further reported that moths in the hypomagnetic conditions had less flight activity late in the night compared to the control group. They suggest that the latter may be related to the circadian rhythm of *M. separata*.

Sarimov *et al.* reported that hypomagnetic conditions influence human cognitive processes [181]. They concluded that exposure to hypomagnetic fields resulted in an increased number of errors and extension of the time required to complete the tasks compared to normal conditions.

Wang and co-workers showed that exposure to hypomagnetic fields induced cell proliferation of SH-SY5Y cells in a glucose-dependent manner [182]. They suggested that lactate dehydrogenase was a direct response to cell proliferation under hypomagnetic conditions. The authors further proposed that the up-regulation of anaerobic glycolysis and repression of oxidative stress shifted cellular metabolism more towards the Warburg effect commonly observed in cancer metabolism. Table 2 summarizes hypomagnetic field effects observed on various physiological functions.

**Table 2.** Hypomagnetic field effects on different biological functions.

system	references
**development**	
decrease in size and number of *Staphylococcus aureus*	Rosenbach [159]
changes of tinctorial, morphological, cultural and biochemical properties in bacteria	Eerkin *et al.* [183]
newt (*Cynops pyrrhogaster*)—early developmental processes	Asashima *et al.* [160]
inhibition of early embryogenesis	Osipenko [161,162]
*Xenopus* embryos—development	Mo *et al.* [163]
*Arabidopsis*—cryptochrome-related hypocotyl growth and flowering	Xu *et al.* [164,165]
brown planthopper—development and reproduction	Wan *et al.* [166]
increased mortality in tardigrades	Erdmann *et al.* [167]
inhibition of anhydrobiotic abilities in tardigrades	Erdmann *et al.* [168]
developmental and behavioural effects in moths	Yan *et al.* [180]
cell proliferation in SH-SY5Y cells, ROS implicated	Wang *et al.* [182]
**circadian system**	
fiddler crabs and other organisms—circadian clock	Brown [169]
human—circadian rhythms	Waver *et al.* [170]
bird—circadian clock	Bliss & Heppner [171]
mice—circadian rhythm/increases algesia	Mo *et al.* [184]
**neurons and brain**	
inhibition of stress-induced analgesia in male mice	Seppia *et al.* [185]
hamster—GABA in cerebellum and basilar nucleus	Junfeng *et al.* [186]
mice—amnesia	Choleris *et al.* [187]
chick—long-term memory	Wang *et al.* [188]
impairment in learning abilities and memory of adult male mice	Wang *et al.* [189]
*Drosophila*—amnesia	Zhang *et al.* [190]
mice—analgesia	Prato *et al.* [191]
golden hamster—noradrenergic activities in the brainstem	Zhang *et al.* [192]
human cognitive processes	Sarimov *et al.* [181]
purified tubulin from calf brain—assembly	Wang *et al.* [173]
chickens needed additional noradrenaline for memory consolidation	Xiao *et al.* [193]
human—cognitive processes	Binhi & Sarimov [194]
human neuroblastoma cell—proliferation	Mo *et al.* [195]
human neuroblastoma cells—actin assembly and inhibits cell motility	Mo *et al.* [196]
human neuroblastoma cell—H_2_O_2_ production	Zhang *et al.* [197]
anxiety in adult male mice	Ding *et al.* [198]
mouse—proliferation of mouse neural progenitor and stem cells	Fu *et al.* [199]
**DNA**	
genetic mutations in *Drosophila* during space flight	Ikenaga *et al.* [175]
mouse ESCs culture—DNA methylation	Baek *et al.* [174]
human bronchial epithelial cells—DNA repair process	Xue *et al.* [200]
**others**	
decreased enzyme activity in cells obtained from mice	Conley [179]
Ca^2+^ balance in meristem cell of pea roots	Belyavskaya [177]
ability to change colour in *Xenopus laevis*	Leucht [201]
chromatin hypercondensation/decondensation in human fibroblasts/lymphocytes	Belyaev *et al.* [178]
increased protoplasts fusion	Nedukha *et al.* [202]
decreasing certain elements in rats’ hair	Tombarkiewicz [203]
cancer-derived cell lines—cell cycle rates	Martino *et al.* [176]
human fibrosarcoma cancer cells—H_2_O_2_ production	Martino *et al.* [204]
mouse primary skeletal muscle cell—ROS levels	Fu *et al.* [205]
invertebrates and fish—calcium-dependent proteases	Kantserova *et al.* [206]

### 2.3. Oscillating magnetic field

#### 2.3.1. Low-frequency

The effects of oscillating magnetic fields on biological functions are abundant [207–215], and are often correlated with modulation of ROS levels [216–218]. In this section, we review several studies on extremely low-frequency (less than 3 kHz) magnetic fields on various biological functions.

Sherrard and co-workers showed that exposure of the cerebellum to low-intensity repetitive transcranial magnetic stimulation (LI-rTMS) (10 mT) modulated behaviour and Purkinje cell morphology [219,220]. Recently, the same group reported that LI-rTMS (2 mT) induced axon growth and synapse formation providing olivocerebellar reinnervation in the cerebellum [221]. The authors concluded that cryptochrome was required for the magnetosensitivity of the neurons, which was consistent with ROS production by activated cryptochrome [222]. In a recent study, the team showed that LI-rTMS (10 mT and 10 Hz) evoked neuronal firing during the stimulation period and induced durable attenuation of synaptic activity and spontaneous firing in cortical neurons of rats *in vivo* [223].

Contalbrigo *et al.* showed that magnetic fields (less than 1 mT, 50 Hz) influenced some haematochemical parameters of circadian rhythms in Sprague–Dawley rats [224]. Further, Fedele *et al.* reported that a 300 μT magnetic field (3–50 Hz) induced changes in two locomotor phenotypes, circadian period and activity levels via modulating cryptochrome in *Drosophila* [225]. Moreover, it has been shown that exposure to a magnetic field of an 0.1 mT and 50 Hz alters clock gene expressions [226].

Manikonda *et al.* applied magnetic fields (50 and 100 μT, 50 Hz) to the cerebellum, hippocampus and cortex of rat brains. They observed that H_2_O_2_ increased in the descending order of cerebellum, hippocampus and cortex. In that work, 100 μT induced more oxidative stress compared to 50 μT [227]. Furthermore, Özgün *et al.* reported that exposure to a magnetic field (1 mT, 50 Hz) *in vitro* induced human neuronal differentiation through *N*-methyl-d-aspartate (NMDA) receptor activation [228]. They observed that the magnetic field enhanced intracellular Ca^2+^ levels. The authors concluded that NMDA receptors (NMDARs) are essential for magnetosensitivity in such phenomena. It is also known that a combination of static (27–37 μT) and time varying (13/114 μT, 7/72 Hz) magnetic fields directly interact with the Ca^2+^ channel protein in the cell membrane [229]. It has also been reported that exposure to greater than 5 mT (50 Hz) magnetic fields may promote X-ray-induced mutations in hamster ovary K1 cells [230]. Koyama *et al.* showed that exposure to a magnetic field of 5 mT (60 Hz) promoted damage induced by H_2_O_2_, resulting in an increase in the number of mutations in plasmids in *E. coli* [231]. Studies of extremely low-frequency magnetic field effects (less than 1000 Hz) on various biological functions are shown in tables 3 and 4.

**Table 3.** Extremely low-frequency (less than 3 kHz) magnetic field effects on memory, stress, pain, dopamine, serotonin, melatonine, genetics and calcium flux.

system	magnetic field and frequency	references
**memory**		
rat—acquisition and maintenance of memory	2 mT, 50 Hz	Liu *et al.* [232]
rat—memory and corticosterone level	0.2 mT, 50 Hz	Mostafa *et al.* [233]
spatial recognition memory in mice	0.6/0.9/1.1/2 mT, 25/50 Hz	Fu *et al.* [234]
spatial memory disorder/hippocampal damage in Alzheimer’s disease rat model	400 μT, 50 Hz	Liu *et al.* [235]
recognition memory task/hippocampal spine density in mice	1 mT, 50 Hz	Zhao *et al.* [236]
human hippocampal slices—semantic memory	1 μT, 5 min on/5 min off	Richards *et al.* [237]
**stress**		
behaviour/anxiety in rats	520 μT, 50 Hz	Balassa *et al.* [238]
benzodiazepine system in hyperalgesia in rats	0.5/1/2 mT, 60 Hz	Jeong *et al.* [239]
anxiogenic effect in adult rats	2 mT, 50 Hz	Liu *et al.* [240]
anxiety level and spatial memory of adult rats	2 mT, 50 Hz	He *et al.* [241]
stress-related behaviour of rats	10 mT, 50 Hz	Korpinar *et al.* [242]
depression and corticosterone secretion in mice	1.5/3 mT, 60 Hz	Kitaoka *et al.* [243]
anxiety, memory and electrophysiological properties of male rats	4 mT, <60 Hz	Rostami *et al.* [244]
induction of anxiety via NMDA activation in mice	1 mT, 50 Hz	Salunke *et al.* [245]
**pain**		
mice—pain thresholds	2 mT, 60 Hz	Jeong *et al.* [246]
snail—analgesia	141−414 μT, 30 & 60 Hz	Prato *et al.* [247]
human—analgesia/EEG	200 μT, <500 Hz	Cook *et al.* [248]
attenuate chronic neuropathic pain in rats	1 mT, 1/10/20/40 Hz	Mert *et al.* [249]
mice—inhibition of morphine-induced analgesia	0.15-9 mT, 0.5 Hz	Kavaliers & Osscnkopp [250]
**dopamine/serotonin/melatonin**		
rat frontal cortex—dopamine and serotonin level	1.8–3.8 mT, 10 Hz	Siero *et al.* [251]
rat brain—serotonin and dopamine receptors activity	0.5 mT, 50 Hz	Janac *et al.* [252]
rat—central dopamine receptor	1.8–3.8 mT, 10 Hz	Siero *et al.* [253]
rat—plasma and pineal melatonin levels	1/5/50/250 μT, 50 Hz	Kato *et al.* [254]
human—melatonin concentration	2.9 mT, 40 Hz	Karasek *et al.* [255]
**genetic**		
rat brain cells—increases DNA strand breaks	0.5 mT, 60 Hz	Lai & Singh [256,257]
human HL-60 cells-steady—state levels of some mRNAs	8 μT, 60 Hz	Karabakhtsian *et al.* [258]
hamster ovary K1cells—promotion in X-ray-induced mutations	>5 mT, 50 Hz	Miyakoshi *et al.* [230]
HL-60 cells—CREB DNA binding activation	0.1 mT, 50 Hz	Zhou *et al.* [259]
plasmids in *E. coli*—increase in the number of mutations	5 mT, 60 Hz	Komaya *et al.* [231]
genetic analysis of circadian responses in *Drosophila*	300 μT, 3–50 Hz	Fedele *et al.* [225]
epigenetic modulation of adult hippocampal neurogenesis in mice	1 mT, 50 Hz	Leone *et al.* [260]
circadian gene expression in human fibroblast cell	0.1 mT, 50 Hz	Manzella *et al.* [226]
epigenetic modulation in human neuroblastoma cells	1 mT, 50 Hz	Consales *et al.* [261]
**calcium**		
lymphocyte—calcium signal transduction	42.1 μT, 16 Hz	Yost & Liburdy [262]
T cell—intracellular calcium oscillations	0.1 mT, 50 Hz	Lindströum *et al.* [263]
rat pituitary cells—Ca^2+^ influx	50 μT, 50 Hz	Barbier *et al.* [264]
Ca^2+^ channel protein in the cell membrane	13/114 μT, 7/72 Hz	Baurus Koch *et al.* [229]
human skin fibroblast populations—intracellular calcium oscillations	8 mT, 20 Hz	Löschinger *et al.* [265]
osteoblasts cells—intracellular calcium levels	0.8 mT, 50 Hz	Zhang *et al.* [266]
C2C12 muscle cells—calcium handling and increasing H_2_O_2_	1 mT, 50 Hz	Morabito *et al.* [267]
rat ventricle cells—intracellular Ca^2+^	0.2 mT, 50 Hz	Sert *et al.* [268]
mesenchymal stem cells—Ca^2+^ intake	1 mT, 50 Hz	Özgün & Garipcan [269]
brain tissue—radiation-induced efflux of Ca^2+^ ions	μT, 15/45 Hz	Blackman *et al.* [270]
rat hippocampus—Ca^2+^ signalling and NMDA receptor functions	50/100 μT, <300 Hz	Manikonda *et al.* [271]
entorhinal cortex neurons—calcium dynamics	1/3 mT, 50 Hz	Luo *et al.* [272]

**Table 4.** Extremely low-frequency (less than 3 kHz) magnetic field effects on reactive oxygen species (ROS) levels.

system	magnetic field	references
**ROS**		
ageing via ROS involvement in brain of mongolian gerbils	0.1/0.25/0.5 mT, 50 Hz	Selakovi *et al.* [273]
hippocampus mitochondria via increasing H_2_O_2_ in mice	8 mT, 50 Hz	Duan *et al.* [274]
neural differentiation/H_2_O_2_ elevation in mesenchymal stem cells	1 mT, 50 Hz	Park *et al.* [275]
H_2_O_2_ production in neuroblastoma cell	2 ± 0.2 mT, 75 ± 2 Hz	Osera *et al.* [276]
pro-Parkinson’s disease toxin MPP^+^/H_2_O_2_ increase in SH-SY5Y cells	1 mT, 50 Hz	Benassi *et al.* [277]
rat peritoneal neutrophils-oxidative burst	0.1 mT, 60 Hz	Roy *et al.* [278]
cortical synaptosomes of Wistar rats-oxidative stress	0.7 mT, 60 Hz	Túnez *et al.* [279]
pro-oxidant effects of H_2_O_2_ in human neuroblastoma cells	2 mT, 75 Hz	Falone *et al.* [280]
reducing hypoxia/inflammation damage ROS-mediated in neuron-like and microglial cells	1.5 ± 0.2 mT, 75 Hz	Vincenzi *et al.* [281]
mouse brain-antioxidant defense system	1.2 mT, 60 Hz	Lee *et al.* [282]
rat-cortical neurons-redox and trophic response/reducing ROS	1 mT, 50 Hz	DiLoreto *et al.* [283]
human monocytes-cell activating capacity/ROS modulation	1 mT, 50 Hz	Lupke *et al.* [284]
HL-60 leukaemia cells-proliferation/DNA damage implicating ROS	1 mT, 50 Hz	Wolf *et al.* [285]
human monocytes-alteration of 986 genes/modulating ROS	1 mT, 50 Hz	Lupke *et al.* [286]
prostate cancer cells-apoptosis through ROS	0.2 mT, 60 Hz	Koh *et al.* [287]
K562 cells-O_2_^·−^ formation and HSP70 induction	0.025–0.1 mT, 50 Hz	Mannerling *et al.* [288]
K562 Cells-differentiation via increasing O_2_^·−^ production	5 mT, 50 Hz	AySe *et al.* [289]
K562 leukaemia cell-number of apoptotic cells via increasing O_2_^·−^ production	1 mT, 50 Hz	Garip & Akan [290]
PC12 cells-H_2_O_2_ increase	1 mT, 50 Hz	Morabito *et al.* [291]
carcinoma cells-cisplatin via increasing H_2_O_2_	1 mT, 50 Hz	Bułdak *et al.* [292]
human carcinoma cells-morphology and biochemistry implicating ROS	0.1 mT, 100&217 Hz	Sadeghipour *et al.* [293]
rats- DNA strand breaks in brain cells by modulating ROS	0.1–0.5 mT, 60 Hz	Lai & Singh [294]
cardiomyocytes-injury treatment implicating ROS	4.5 mT, 15 Hz	Ma *et al.* [295]
genomic instability/oxidative processes in human neuroblastoma cells	100 μT, 50 Hz	Luukkonen *et al.* [296]
expression of NOS and O_2_^·−^ in human SH-SY5Y cells	1 mT, 50 Hz	Reale *et al.* [297]
ROS-related autophagy in mouse embryonic fibroblasts	2 mT, 50 Hz	Chen *et al.* [298]
healing via reducing ROS production in artificial skin wounds	<40 μT, 100 Hz	Ferroni *et al.* [299]
apoptosis via oxidative stress in human osteosarcoma cells	1 mT, 50 Hz	Yang *et al.* [300]
increase O_2_^·−^ in erythro-leukemic cells	1 mT, 50 Hz	Patruno *et al.* [301]
Genomic instability/H_2_O_2_ increase in SH-SY5Y cells	100 μT, 50 Hz	Kesari *et al.* [302]
NOX-produced ROS in hAECs	0.4 mT, 50 Hz	Feng *et al.* [303]
mitochondrial permeability via increasing H_2_O_2_ in human aortic endothelial cells	0.4 mT, 50 Hz	Feng *et al.* [304]
apoptotic via mitochondrial O_2_^·−^ release in human aortic endothelial cells	0.4 mT, 50 Hz	Feng *et al.* [305]
antioxidant activity implicating H_2_O_2_ in human keratinocyte cells	25 − 200 μT, 1–50 Hz	Calcabrini *et al.* [306]
antioxidative defense mechanisms via ROS in human osteoblasts	2 − 282 μT, 16 Hz,	Ehnert *et al.* [307]
astrocytic differentiation implicating ROS in human bone stem cells	1 mT, 50 Hz	Jeong *et al.* [308]
reduce mitochondrial O_2_^·−^ production in human neuroblastoma cells	100 μT, 50 Hz	Höytö *et al.* [309]
ROS production in human cryptochrome	1.8 mT, <100 Hz	Sherrard *et al.* [222]
proliferation by decreasing intracellular ROS levels in human cells	10 mT, 60 Hz	Song *et al.* [310]
cytotoxic effect in by raising intracellular ROS in human GBM cells	1–58 mT, 350 Hz	Helekar *et al.* [311]

#### 2.3.2. Medium/high-frequency

In this section, we review several studies on medium/high-frequency (greater than 3 kHz) magnetic field effects on various physiological functions (table 5). Usselman *et al.* reported that oscillating magnetic fields at Zeeman resonance (1.4 MHz and 50 μT) influenced relative yields of cellular O_2_^·−^ and H_2_O_2_ products in human umbilical vein endothelial cells [340]. Considering a radical pair in [FADH^.^..O_2_^·−^] form, the authors suggested that coherent electron spin dynamics may explain their observation. Moreover, Friedman *et al.* observed that a 875 MHz magnetic field increased ROS production, which was mediated by membrane-associated NOX in HeLa cells and rats [341]. Castello and colleagues showed that exposure of fibrosarcoma HT1080 cells to weak radio frequency (5/10 MHz) combined with a 45 μT static magnetic field modulated the number of cells and significantly increased H_2_O_2_ production [342]. Martino and Castello showed that exposure of cultured yeast and isolated mitochondria to magnetic fields (150 μT; 45 μT and a parallel 10 MHz RF; 45 μT and a perpendicular 10 MHz RF) modulated the production of extracellular, intracellular, and mitochondrial O_2_^·−^ and H_2_O_2_ [343]. They concluded that complex I of the ETC is involved in H_2_O_2_ production. Table 6 summarizes a few medium/high-frequency magnetic field effects observed in various experiments.

**Table 5.** Extremely low-frequency (less than 3 kHz) magnetic field effects on different biological functions.

system	magnetic field	references
**others**		
neuroendocrine cell—proliferation and death	<1 mT, 50 Hz	Grassi *et al.* [312]
cortices of mice—neuronal differentiation of neural stem/progenitor cells	1 mT, 50 Hz	Piacentini *et al.* [313]
hippocampal slices—excitability in hippocampal neurons	15 mT, 0.16 Hz	Ahmed & Wieraszko [314]
human—EEG alpha activity	200 μT, 300 Hz	Cook *et al.* [315,316]
rat—neuroprotective effects	0.1/0.3/0.5 mT, 15 Hz	Yang *et al.* [317]
rat—neuroprotective effects on Huntington’s disease	0.7 mT, 60 Hz	Tasset *et al.* [318]
synaptic efficacy in rat brain slices	0.5/3 mT, 50 Hz	Balassa *et al.* [319]
global cerebral ischaemia/pituitary ACTH and TSH cells in gerbils	0.5 mT, 50 Hz	Balind *et al.* [320]
neurotrophic factor expression in rat dorsal root ganglion neurons	1 mT, 50 Hz	Li *et al.* [321]
visual cortical circuit topography and BDNF in mice	∼10 mT, <10 Hz	Makowiecki *et al.* [322]
hippocampal long-term potentiation in rat	100 μT, 50 Hz	Komaki *et al.* [323]
neuronal GABAA current in rat cerebellar granule neurons	1 mT, 50 Hz	Yang *et al.* [324]
central nervous regeneration in planarian *Girardia sinensis*	200 mT, 60 Hz	Chen *et al.* [325]
neuronal differentiation and neurite outgrowth in embryonic neural stem cells	1 mT, 50 Hz	Ma *et al.* [326]
synaptic transmission and plasticity in mammalian central nervous synapse	1 mT, 50 Hz	Sun *et al.* [327]
human—pineal gland function	<μT, 60 Hz	Wilson *et al.* [328]
rat—electrically kindled seizures	0.1 mT, 60 Hz	Ossenkopp & Cain [329]
rat—central cholinergic systems	1 mT, 60 Hz	Lai *et al.* [330]
deer mice—spatial learning	0.1 mT, 60 Hz	Kavaliers *et al.* [331]
T-cell receptor—signalling pathway	0.15 mT, 50 Hz	Lindström *et al.* [332]
enhances locomotor activity via activation of dopamine D1-like receptors in mice	0.3/2.4 mT, 60 Hz	Shin *et al.* [333]
rat pituitary ACTH cells	0.5 mT, 50 Hz	Balind *et al.* [334]
actin cytoskeleton reorganization in human amniotic cells	0.4 mT, 50 Hz	Wu *et al.* [335]
reduces hypoxia and inflammation in damage microglial cells	1.5 mT, 50 Hz	Vincenzi *et al.* [281]
pluripotency and neuronal differentiation in mesenchymal stem cells	20 mT, 50 Hz	Haghighat *et al.* [336]
proliferation and differentiation in osteoblast cells	5 mT, 15 Hz	Tong *et al.* [337]
reduced hyper-inflammation triggered by COVID-19 in human	10 mT, 300 Hz	Pooam *et al.* [338]
proliferation and regeneration in planarian *Schmidtea mediterranea*	74 μT, 30 Hz	Ermakov *et al.* [339]

**Table 6.** Medium/High-frequency (greater than 3 kHz) magnetic field effects on biological functions.

system	magnetic field and frequency	references
ROS production and DNA damage in human SH-SY5Y neuroblastoma cells	872 MHz	Luukkonen *et al.* [344]
ROS level in human ejaculated semen	870 MHz	Agarwal *et al.* [345]
ROS production and DNA damage in human spermatozoa	1.8 GHz	Iuliis *et al.* [346]
ROS levels and DNA fragmentation in astrocytes	900 MHz	Campisi *et al.* [347]
ROS formation and apoptosis in human peripheral blood mononuclear cell	900 MHz	Lu *et al.* [348]
ROS elevation in *Drosophila*	1.88–1.90 GHz	Manta *et al.* [349]
ROS modulation in rat pulmonary arterial smooth muscle cells	7 MHz	Usselman *et al.* [350]
bioluminescence and oxidative response in HEK cells	940 MHz	Sefidbakht *et al.* [351]
electrical network activity in brain tissue	<150 MHz	Gramowski-Voß *et al.* [352]
ROS production in human umbilical vein endothelial cells	50 μT, 1.4 MHz	Usselman *et al.* [340]
insect circadian clock	420 μT, RF	Bartos *et al.* [353]
tinnitus, migraine and non-specific in human	100 KHz to 300 GHz	Röösli *et al.* [354]
magnetic compass orientation in night-migratory songbird	75–85 MHz	Leberecht *et al.* [355]

### 2.4. Isotope effects

Atomic nuclei contain protons and neutrons. The number of protons determines the element (e.g. carbon, oxygen etc.), and the number of neutrons determines the isotope of the desired element. Some isotopes are stable, i.e. they preserve the number of protons and neutrons during chemical reactions. It has been shown that using different isotopes of the element in certain chemical reactions results in different outcomes. Such observations have been seen in many chemical reactions [356–363] including biological processes [45,364–368]. Inheriting quantum properties, not only do different isotopes of an element have different masses, but they can also have different spins. For that reason, isotope effects in (bio)chemical reactions can be regarded from two distinct points of view: mass-dependency and spin-dependency. Thiemens *et al.* observed mass-independent isotope effects as a deviation of isotopic distribution in reaction products [369–373]. Furthermore, in 1976 Buchachenko and colleagues by applying magnetic fields detected the first mass-independent isotope effect, which chemically discriminated isotopes by their nuclear spins and nuclear magnetic moments [374]. Since then, the term ‘magnetic isotope effect’ was dubbed for such phenomena as they are controlled by electron-nuclear hyperfine coupling in the paramagnetic species. Moreover, isotope effects have been observed for a great variety of chemical and biochemical reactions involving oxygen, silicon, sulfur, germanium, tin, mercury, magnesium, calcium, zinc and uranium [65,367,368,375–381]. In this review, we focus on isotope effects from a spin perspective, see table 7.

**Table 7.** Spin-dependent isotope effects on different biological functions.

system	isotope	spin, *I*	references
parenting/offspring development in rat	^6^Li, ^7^Li	1, 3/2	Sechzer *et al.* [382]
hyperactivity in rat	^6^Li, ^7^Li	1, 3/2	Ettenberg *et al.* [383]
anaesthetic potency in mice	^129^Xe, ^131^Xe, ^132^Xe, ^134^Xe	1/2, 3/2, 0, 0	Li *et al.* [384]
ATP production in purified pig skeletal muscle PGK	^24^Mg, ^25^Mg, ^26^Mg	0, 5/2, 0	Buchachenko *et al.* [385]
DNA synthesis in HL-60 human myeloid leukaemia cells	^64^Zn, ^67^Zn	0, 5/2	Buchachenko *et al.* [386]
DNA synthesis in HL-60 human myeloid leukaemia cells	^24^Mg, ^25^Mg, ^26^Mg	0, 5/2, 0	Buchachenko *et al.* [387]
DNA synthesis in HL-60 human myeloid leukaemia cells	^40^Ca, ^43^Ca	0, 7/2	Bukhvostov *et al.* [388]

In 1986 Sechzer and co-workers reported that lithium administration results in different parenting behaviours and potentially delayed offspring development in rats [382]. Their findings were not quantitative; however, it was observed that different lithium isotopes exhibited different impacts. Moreover, in 2020, Ettenberg *et al.* [383] conducted an experiment demonstrating an isotope effect of lithium on rat hyperactivity. Lithium has two stable isotopes, ^6^Li and ^7^Li, possessing different nuclear spin angular momentum, *I*_6_ = 1 and *I*_7_ = 3/2, respectively. In that work, the mania phase was induced by sub-anaesthetic doses of ketamine. The authors reported that ${\;}^{6}\mathrm{Li}$ produced a longer suppression of hyperactivity in an animal model of mania compared to ^7^Li. We further discuss this phenomenon from the point of view of the radical pair mechanism in §4.2.

Li and co-workers reported that xenon (Xe)-induced anaesthesia in mice is isotope-dependent. They used four different Xe isotopes, ^129^Xe, ^131^Xe, ^132^Xe and ^134^Xe with nuclear spins of 1/2, 3/2, 0 and 0, respectively [384]. The results fell into two groups, isotopes with spin and isotopes without spin, such that isotopes of xenon with non-zero nuclear spin had lower anaesthetic potency than isotopes with no nuclear spin. The results of this work are discussed from the perspective of the radical pair mechanism in §4.1.

Buchachenko *et al.* observed that magnesium-25 (^25^Mg) controlled phosphoglycerate kinase (PGK) [385]. ^25^Mg has a nuclear spin of 5/2, while ^24^Mg is spin-less. The authors reported that ATP production was more than twofold in the presence of ^25^Mg compared to ^24^Mg. They suggested that the nuclear spin of Mg was the key factor for such an observation. In another study, the same group reported that ^25^Mg reduced enzymatic activity in DNA synthesis compared to ^24^Mg. They concluded that DNA synthesis is magnetic field-dependent [387,389]. In the same system, they further observed that if Mg^2+^ ion is replaced by stable isotopes of calcium ion, ^40^Ca^2+^ and ^43^Ca^2+^ (with nuclear spins of 0, 7/2, respectively), the enzyme catalytic reactions will be isotope-dependent, such that ^43^Ca^2+^promoted enzyme hyper-suppression leading to a residual synthesis of shorted DNA fragments compared to ^40^Ca^2+^ [388]. They repeated the same experiment but this time instead of Mg^2+^ ion stable isotopes of zinc, ^64^Zn^2+^ and ^67^Zn^2+^ (with nuclear spins of 0, 5/2, respectively) were used. The authors reported that ^67^Zn^2+^ suppressed DNA synthesis a few times more than ^64^Zn^2+^ [386].

## 3. The radical pair mechanism

### 3.1. Spin and radical pairs

Spin is an inherently quantum property that emerges from Dirac’s relativistic quantum mechanics [390,391], and is described by two numbers, *S* and *m*_*s*_, respectively, the spin quantum number and the spin projection quantum number. Electrons, protons and neutrons have spins of *S* = 1/2. Having an angular momentum characteristic, spin can be coupled not only with external magnetic fields but also with other spin in its vicinity. For instance, coupling of two electrons spins, **S**_*A*_ and **S**_*B*_, results in a total spin of **S**_*T*_, which has a quantum number of either *S* = 1 or *S* = 0. The latter case is called a singlet state, with *m*_*s*_ = 0, and the former is called a triplet state, with *m*_*s*_ = 0, ±1 [392].

3.1 $$|S\rangle =\frac{1}{\sqrt{2}}({\left|↑\right\rangle }_{A}⊗{\left|↓\right\rangle }_{B}-{\left|↓\right\rangle }_{A}⊗{\left|↑\right\rangle }_{B}),$$

3.2 $$\left|T_{-}\right\rangle ={\left|↓\right\rangle }_{A}⊗{\left|↓\right\rangle }_{B},$$

3.3 $$\left|T_{0}\right\rangle =\frac{1}{\sqrt{2}}({\left|↑\right\rangle }_{A}⊗{\left|↓\right\rangle }_{B}+{\left|↓\right\rangle }_{A}⊗{\left|↑\right\rangle }_{B})$$

3.4 $$\;\mathrm{and}\;\;\left|T_{+}\right\rangle ={\left|↑\right\rangle }_{A}⊗{\left|↑\right\rangle }_{B},$$

where ⊗ is the tensor product.

Radicals are molecules with an odd number of electrons in the outer shell [393,394]. A pair of radicals can be formed by breaking a chemical bond or electron transfer between two molecules. It is important to note that in reactions of organic molecules, spin is usually a conserved quantity, which is essential for magnetic field effect in biochemical reactions. For example, a radical pair can be created if a bond between a pair of molecules [A · · · D] breaks or an electron is transferred from D to A, [A^−.^ · · · D^.+^] (D and A denote donor and acceptor molecules). A radical pair may be in a superposition of singlet and triplet states, depending on the parent molecule’s spin configuration. Assuming that the initial state of the electron pairs before separation was a singlet (triplet), the recombination of unpaired electrons can only happen if they stayed in a singlet (triplet) [395].

If the radical pairs are formed in singlet (triplet) states, the initial spin density matrix reads as follows:

3.5 $$\hat{\rho }(0)=\frac{1}{M}{\hat{P}}^{S},$$

3.6 $${\hat{P}}^{S}=\left|S\right\rangle ⊗\left\langle S\right|⊗{\hat{𝟙}}_{M},$$

3.7 $${\hat{P}}^{T}=\{\left|T_{+}\right\rangle ⊗\left\langle T_{+}\right|+\left|T_{0}\right\rangle ⊗\left\langle T_{0}\right|+\left|T_{-}\right\rangle ⊗\left\langle T_{-}\right|\}⊗{\hat{𝟙}}_{M},$$

3.8 $${\hat{P}}^{S}+{\hat{P}}^{T}={\hat{𝟙}}_{4M}$$

3.9 $$\;\mathrm{and}\;\;M=\prod_{i}^{n}(2I_{i}+1),$$

where ${\hat{P}}^{S}$ and ${\hat{P}}^{T}$ are the singlet and triplet projection operators, respectively, *M* is the nuclear spin multiplicity, *I*_*i*_ is the spin angular momentum of *i*th nucleus and $\hat{𝟙}$ is the identity matrix. S is entangled. The T projector is not entangled, even though |*T*_0_〉 is an entangled state.

### 3.2. Interactions

#### 3.2.1. Zeeman interaction

The interaction between the unpaired electron spins on each radical and the external magnetic field is essential for generating MFEs. This interaction is called the Zeeman effect [396]. The nuclear spins of radical molecules also experience applied magnetic fields; however, as nuclear magnetogyric ratios are much smaller than that of the electrons, these interactions are negligible. The Zeeman interaction is defined in the following form:

3.10 $${\hat{H}}_{Z}=\mu_{B}\hat{S}.g.B,$$

where μ_*B*_, $\hat{S}$, **g**-tensor and **B** are the Bohr magneton, the spin operators of electron, the interaction coupling and applied magnetic field, respectively. Here, we focus on magnetic field interactions with relatively low field strengths. In such cases, it is possible to assume that the **g**-tensor equals to *g*_*e*_ of free electron, and hence,

3.11 $${\hat{H}}_{Z}=g_{e}\mu_{B}\hat{S}.B=-\gamma_{e}h\hat{S}.B,$$

where *γ*_*e*_ and *h* are the electron magnetogyric ratio and the Planck constant, respectively.

#### 3.2.2. Hyperfine interaction

Similar to electron–electron spin coupling, electron spins can couple to the nuclear spins, called hyperfine interactions [397]. This interaction consists of two contributions, isotropic and anisotropic interactions. The former is also called Fermi contact term, which results from the magnetic interaction of the electron and nuclear spins when the electron is *within* the nucleus. The overall hyperfine interaction can be defined as follows:

3.12 $${\hat{H}}_{HFI}=\hat{S}.a_{i}.{\hat{I}}_{i},$$

where **a**_*i*_ and ${\hat{I}}_{i}$ are the hyperfine coupling tensor and nuclear spin of *i*th nucleus. The anisotropic components of the hyperfine interactions are only relevant when the radicals are immobilized and aligned [25]. Neglecting the anisotropic component of the hyperfine interaction, the hyperfine Hamiltonian has the following form:

3.13 $${\hat{H}}_{HFI}=a_{i}\hat{S}.{\hat{I}}_{i},$$

where *a*_*i*_ is the isotropic hyperfine coupling constant and can be calculated as

3.14 $$a_{i}=-\frac{2}{3}g_{e}\gamma_{e}\gamma_{n}\mu_{0}|\Psi (0){|}^{2},$$

*μ*_0_ is the vacuum permeability, *γ*_*n*_ is the nuclear magnetogyric ratio and $|\Psi (0){|}^{2}$ is the electron probability density at the nucleus [398].

#### 3.2.3. Exchange interaction

The electrons on radicals are identical in quantum calculations. This indistinguishability of electrons on radical pairs can be introduced via the exchange interaction [399]. It is generally assumed to weaken exponentially with increasing radical pair separation. The exchange interaction can prevent singlet–triplet interconversion, as discussed later. However, recent studies show that this term is negligible [400] in the magnetic field effects on pigeon cryptochrome [401].

#### 3.2.4. Dipolar interaction

As spins are magnetic moments, the radical pairs also influence each other by a dipolar interaction [402]. This interaction can suppress singlet–triplet interconversion in the radical pair dynamics. However, studies on avian magnetoreception suggest that under certain conditions exchange and dipolar interactions can be neglected [43,403–406].

#### 3.2.5. Other contributions

It is thought that after a first re-encounter, radicals either react or diffuse apart forever [407]. In the context of birds’ magnetoreception, for this contribution, an exponential model is used [43,408].

High electron density on an atom of a radical can lead to have a higher anisotropic *g*-value compared to the case with lower electron density, called the spin-orbit effect, which results in the non-radiative transition between two electronic states with different spin multiplicity (e.g. singlet and triplet)—intersystem crossing, which can play important roles in chemical reactions [409–412].

### 3.3. Spin dynamics of radical pairs

The sensitivity of certain reactions to weak magnetic fields relies on the oscillations between singlet and triplet states of radical pairs, also known as ‘quantum beats’ [26]. If the radicals are separated enough spatially, having the same energies, singlet and triplet will undergo a coherent interconversion process, quantum beating. The interconversion is tuned by the magnetic fields experienced by the electrons, including Zeeman and hyperfine interactions. At low magnetic fields, the main drive for S–T interconversion is due to the hyperfine interactions. Obeying selection rules, the singlet and triplet yields will follow different chemical pathways, which depend on the timing of the coherent spin dynamics [413]. These quantum beats have just recently been observed directly [414].

The fractional singlet yield resulting from the radical pair mechanism throughout the reaction can be normally defined by using the Liouville–von Neumann equation [50]

3.15 $$\frac{d\hat{\rho }(t)}{dt}=-\frac{i}{\hbar }[\hat{H},\hat{\rho }(t)],$$

where $\hat{\rho }(t)$ and $\hat{H}$ are the spin density and Hamiltonian operators, respectively. [ · , · ] denotes the commutator.

For instance, the probability of finding the radical pairs in singlet states at some later time is determined by Hamiltonian using equation (3.15)

3.16 $$\langle {\hat{P}}^{S}\rangle (t)=\mathrm{Tr}[{\hat{P}}^{S}\hat{\rho }(t)],$$

where Tr is trace.

The probability $\left\langle {\hat{P}}^{S}\rangle (t\right)$ depends on other contributions, including kinetic reactions, spin relaxation, vibration and rotation of radical pairs, which can be introduced to equation (3.15).

#### 3.3.1. Static magnetic field

Static magnetic field effects have been extensively studied in the context of birds’ magnetosensitivity [46,48]. However, the applications of these models can be extended to other magnetic field effects reviewed in §2.1. Assuming that the spin of the radical pairs start off from a singlet state, equation (3.16) can be rewritten as

3.17 $$\langle {\hat{P}}^{S}\rangle (t)=\frac{1}{M}\sum_{m}^{4M}\sum_{n}^{4M}|\left\langle m\right|{\hat{P}}^{S}\left|n\right\rangle {|}^{2}\cos\left([\omega_{m}-\omega_{n}]t\right),$$

where |*m*〉 and |*n*〉 are eigenstates of $\hat{H}$ with corresponding eigenenergies of *ω*_*m*_ and *ω*_*n*_, respectively.

Spin relaxation can be introduced phenomenologically [408,415] such that

**Figure 1.**
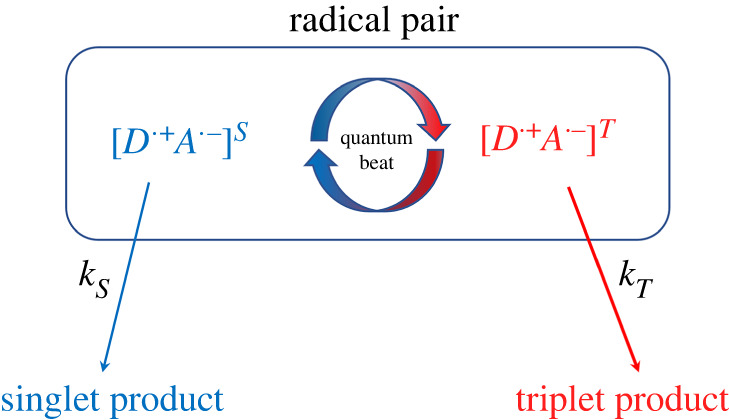
A simple schematic presentation of donor (*D*)–acceptor (*A*) radical pair reaction undergoing intersystem crossing between singlet (*S*) and triplet (*T*) states. Each state takes different chemical pathways via distinct reaction rates to produces *S* and *T* products with *k*_*S*_ and *k*_*T*_, respectively, for *S* and *T* states.

3.18 $$\langle {\hat{P}}^{S}\rangle (t)⟶\frac{1}{4}-\left(\frac{1}{4}-\langle {\hat{P}}^{S}\rangle (t)\right)\;e^{-rt},$$

where *r* denotes the spin relaxation rate. Following the work of Timmel *et al.* [50], the chemical fate of the radical pair can be modelled separating spin-selective reactions of the singlet and triplet pairs, as shown in figure 1. For simplicity, it is assumed that *k* = *k*_*S*_ = *k*_*T*_, where *k*_*S*_ and *k*_*T*_ are the singlet and triplet reaction rates, respectively. The final singlet yield, $\Phi_{S}$, for periods much greater than the radical pair lifetime reads as follows:

3.19 $$\begin{array}{rl} \Phi_{S}=\; & k\int_{0}^{\infty }\langle {\hat{P}}^{S}\rangle (t)\;e^{-kt}\;dt=\;\frac{1}{4}-\frac{k}{4(k+r)}\\ & +\frac{1}{M}\sum_{m}^{4M}\sum_{n}^{4M}|\left\langle m\right|{\hat{P}}^{S}\left|n\right\rangle {|}^{2}\frac{k(k+r)}{{\left(k+r\right)}^{2}+{\left(\omega_{m}-\omega_{n}\right)}^{2}}, \end{array}$$

where the fractional triplet yield can be calculated as $\Phi_{T}=1-\Phi_{S}$.

In §4, we briefly review recent studies that suggest the radical pair mechanism may explain xenon-induced anaesthesia, lithium effects on hyperactivity, magnetic field and lithium effects on circadian clock, and hypomagnetic field effects on neurogenesis and microtubule reorganization. In these studies, for simplicity, only Zeeman and isotropic hyperfine interactions are considered. For a pair of radicals, the Hamiltonian reads

3.20 $$\hat{H}=\omega {\hat{S}}_{A_{z}}+{\hat{S}}_{A}.\sum_{i}^{N_{A}}a_{A_{i}}{\hat{I}}_{A_{i}}+\omega {\hat{S}}_{D_{z}}+{\hat{S}}_{D}.\sum_{i}^{N_{D}}a_{D_{i}}{\hat{I}}_{D_{i}},$$

where ${\hat{S}}_{A}$ and ${\hat{S}}_{D}$ are the spin operators of radical electrons on A^·−^ and D^.+^, respectively, ${\hat{I}}_{A}$ and ${\hat{I}}_{D}$ are the nuclear spin operators on the acceptor and donor radical molecule, *a*_*A*_ and *a*_*B*_ are the isotropic hyperfine coupling constants, *N*_*A*_ and *N*_*D*_ are the number of nuclei coupled to electron *A* and *D*, respectively, and *ω* is the Larmor precession frequency of the electrons due to the Zeeman effect.

#### 3.3.2. Hypomagnetic field

Although hypomagnetic fields belong to the static magnetic field category, the effects due to extremely low magnetic field are often particularly significant compared to other magnetic field effects.

**Figure 2.**
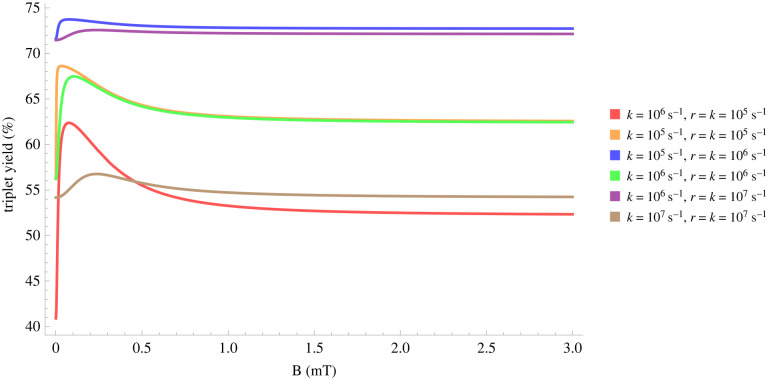
Triplet yield vs applied magnetic field for different reaction and spin relaxation rates for a simple model of a radical pair. In this model, one of the radicals is coupled to a nucleus with a hyperfine coupling constant of 1 mT. For different values of the rates, one can see a pronounced dip near zero field, together with a maximum close to the value of the geomagnetic field (around 0.05 mT)

Using equation (3.19), it can be shown that for different relaxation and reactions rates, the hypomagnetic field effects are significant, as shown in figure 2.

#### 3.3.3. Extremely low-frequency magnetic field

Given the short lifetime of radical pairs compared to the low frequency of the applied magnetic field, in general, the extremely low-frequency magnetic field can be treated as static during the lifetime of a radical pair [408,416]. Depending on the phase of oscillation, *α* ∈ (0, *π*), each radical pair therefore experiences a different, effectively static, magnetic field whose field strength is *B*. Assuming that *B*_0_ and *B*_1_(*t*) are parallel, the net effect of the oscillating field is an average over *α*, such that

3.21 $$B(t)=B_{0}+B_{1}(t)⟹B(t)\equiv B=B_{0}+B_{1}\cos\alpha$$

and

3.22 $$\bar{\Phi_{S}(B_{0},B_{1})}=\frac{1}{\pi }\int_{0}^{\pi }\Phi_{S}(B)\;d\alpha ,$$

where *B*_0_ and *B*_1_ indicate the static magnetic field and the amplitude of the oscillating magnetic field, respectively. Such theoretical model can be applied to the magnetic field effects reviewed in §2.3.1.

#### 3.3.4. Medium/high-frequency magnetic field

For the cases of medium/high-frequency magnetic fields, a general approach is to integrate equation (3.15), using, for example, a fourth-order Runge–Kutta scheme. It is shown that high-frequency magnetic effects can be accounted for by the radical pair mechanism [417–419]. For instance, if the magnetic field has the following form:

3.23 $$B(t)=B_{0}\hat{k}+B_{1}[\cos\omega t\;\hat{i}+\sin\omega t\hat{j}],$$

the corresponding Hamiltonian can be transformed into a rotating reference frame where it becomes a time-independent Hamiltonian [420]. To do so, one could use a unitary transformation matrix

3.24 $$T(t)=e^{i({\hat{S}}_{A_{z}}+{\hat{I}}_{A_{z}}+{\hat{S}}_{B_{z}}+{\hat{I}}_{B_{z}})\omega t},$$

such that

3.25 $$H^{{\;}^{\prime }}=i\hbar \dot{T}(t)T^{-1}(t)+T(t)H(t)T^{-1}(t),$$

Where *H*^′^ is the time-independent Hamiltonian and $\dot{T}(t)$ is the time derivative of *T*(*t*). After some algebra, one can obtain

3.26 $$H^{\text{'}}=g\mu_{B}\sum_{\;j=1}^{2}(B_{0}S_{\;jz}+B_{1}S_{\;jx}+a_{j}{\hat{S}}_{j}.{\hat{I}}_{j})-\omega \sum_{\;j=1}^{2}(S_{\;jz}+I_{\;jz}).$$

### 3.4. Candidate radical pairs

It is now well known that in biology electron-transfer reactions can take place at reasonable rates even when the reactants are separated far beyond ‘collisional’ distances [421,422]. A radical pair can be formed by breaking a chemical bond or electron transfer between two molecules. Electron transfer between proteins is facilitated by the formation of a complex of the reacting proteins, which may be accompanied by conformational changes in the proteins. For that, the reactants must reach each other to build up the coupling of their electronic orbitals. The most used approach to rationalize and predict the rate of electron transfer processes is Marcus electron transfer theory [423]. Determining realistic radical pair candidates for the magnetosensitivity of physiological function, however, is still an interesting challenge. Here, we briefly review a few plausible radical pairs that maybe be relevant for the magnetosensitivity in biology.

#### 3.4.1. Cryptochrome-based radical pairs

In the context of songbird avian magnetoreception, the cryptochrome proteins are the canonical magnetosensitive agent [48,424,425]. Cryptochromes are classified as flavoproteins. They play an important role in the circadian clock, where the circadian function can be either light-dependent or -independent. Kutta *et al.* showed that Type II animal cryptochromes lack the structural features to securely bind the photoactive flavin cofactor [426]. The circadian clock regulates photoreceptor sensitivity in the compound eye of insects and retinas of vertebrates, potentially including the sensitivity of specialized photo-magnetoreceptors. In flies, photo-magnetoreceptors are likely to be an unusual class of photoreceptors, i.e. retinula R7y cells [427]. It is thought that, in cryptochromes and photolyases, photoreduction of FAD is through three consecutive electron transfers along a conserved triad of tryptophan (Trp) residues to give FAD^·−^ and TrpH^.+^ approximately 2 nm distant from each other [428–431]. In cryptochrome-4a, sequentially four radical pair states are formed by the progressive transfer of an electron along a chain of four tryptophan residues to the photo-excited flavin. In a recent study, Hore and co-workers suggest that, based on spin dynamics, while the third radical pair is mainly responsible for magnetic sensing, the fourth could enhance initiation of magnetic signalling particularly if the terminal tryptophan radical can be reduced by a nearby tyrosine (Tyr) [432]. They concluded that this arrangement may play an essential role in sensing and signalling functions of the protein. It is also suggested that Tyr can be the donor instead of the fourth Trp [429]. It is also found based on spin dynamics analysis that a radical pair in the form of [FAD^·−^ and Tyr^.^] can provide sensitivity to the direction of the magnetic field [433].

Alternative radical pairs to [FAD^·−^ · · · TrpH^.+^] have been suggested. In 2009, Ritz and Schulten showed that exposure to low-intensity oscillating magnetic fields disoriented European robins [434]. Interestingly the frequency of the applied magnetic field in that experiment was equal to the Larmor frequency (approx. 1.4 MHz) of a free electron spin in the geomagnetic field. Magnetic fields with the same amplitude but different frequencies had much less impact on the birds’ magnetic compass. Theoretical analysis suggests that such phenomenon may be explained if one of the radicals were free from internal magnetic interactions [435–438], which implies that such an observation is not compatible with the radical pair model based on [FAD^·−^ · · · TrpH^.+^]. Various authors have suggested that the superoxide radical is the most plausible radical under such circumstances [434,435,439–443]; this is also consistent with animal magnetoreception in the dark [444–446], as it was suggested that during the backreaction, a radical pair is formed between flavin and an O_2_ and that the radical pair reaction responds significantly to reorientation in the geomagnetic field [438,439,447–449]. Such a radical pair could be generated without further absorption of light in the form of $\left[{\mathrm{FADH}}^{.}\cdots O_{2}^{.-}\right]$. However, deciding the more realistic radical pair between $\left[{\mathrm{FADH}}^{.}\cdots O_{2}^{.-}\right]$ and [FAD^·−^ · · · TrpH^.+^] to explain avian magnetoreception is still a matter of active debate [446,450–452]. The radical pair involving superoxide demands more reliable evidence.

#### 3.4.2. Beyond cryptochrome-based radical pairs

**Figure 3.**
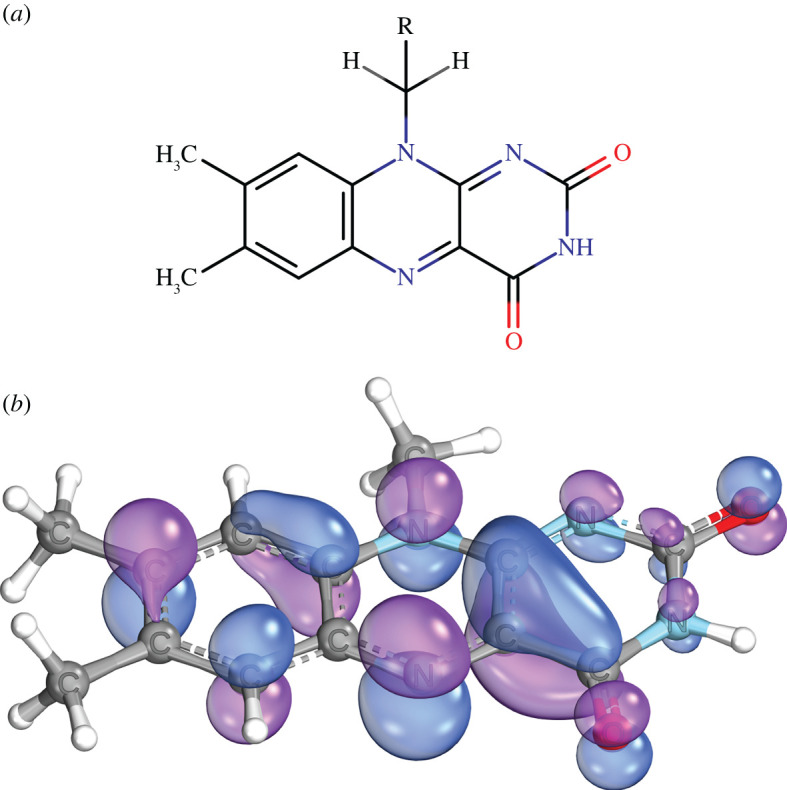
Molecular structure and orbitals of the flavin radical. (*a*) Structure of flavin adenine dinucleotide (FAD). R denotes the adenosine diphosphate group and the rest of the ribityl chain. (*b*) Representations of the molecular orbitals that contain the unpaired electron in a flavin anion radical. Blue and purple indicate parts of the wave function with opposite signs. ORCA package used to calculate the HOMO using PBE0/def2-TZVP [453]. Image rendered using IboView [v20211019-RevA].

Flavin-dependent enzymes are ubiquitous in biology. The isoalloxazine ring of the flavin cofactor (figure 3) can undergo thermally driven redox chemistry. The different redox states of flavin play essential roles in various electron transfer processes and consequently are crucial for a variety of important biological functions, including energy production, oxidation, DNA repair, RNA methylation, apoptosis, protein folding, cytoskeleton dynamics, detoxification, neural development, biosynthesis, the circadian clock, photosynthesis, light emission and biodegradation [422,454–465]. Different forms of transient radical pair intermediates can be created during reactions catalysed by flavin-dependent enzymes, including $\left[{\mathrm{FADH}}^{.}\cdots O_{2}^{.-}\right]$ [466–468].

Although cryptochrome is the main protein for avian magnetoreception, there exist many observational challenges for the canonical cryptochrome-centric radical pair mechanism. In a recent work, Bradlaugh and co-workers observed that the FAD binding domain and the Trp chain in cryptochrome are not required for magnetic field responses at the single neuron and organismal level in *Drosophila*. They further reported that an increase in FAD intracellular concentration enhanced neuronal sensitivity to blue light in the presence of a magnetic field. The authors concluded that the magnetosensitivity in cells may be well explained based on non-cryptochrome-dependent radical pair models [117]. However, the question whether fruit flies use a magnetic compass demands more experimental evidence.

It is known that near the tetrodotoxin binding site in Na^+^ channels there are tryptophan residues. Similarly, in the pore-forming region of voltage-sensitive Na^+^ channels, Tyr and tryptophan residues are located. It is suggested that gating these channel proteins may depend on the electron transfer between these residues, and hence formation of radicals [469]. This form of electron transfer is also proposed to play a key role in DNA photolyase [470].

Many physiological and pathological processes involve protein oxidation [471], icluding important residues such as Trp, Tyr, histidine (His) and proline (Pro). It is known that a radical pair in the form of $\left[{\mathrm{TyrO}}^{.}\cdots O_{2}^{.-}\right]$ can be created [472]. The superoxide radical may also be formed in a spin correlated manner with other partners, including tetrahydrobiopterin [473–475]. In addition, it was shown that an electron transfer process can occur between Trp and superoxide [476,477]. However, as discussed above, the radical pairs involving superoxide is a matter of debate. It was also suggested that in PGK phosphorylation a radical pair [RO^.^ · · · Mg(H_2_O)n^.+^] complex can be formed [385].

## 4. Studies of the potential role of radical pairs in the brain

In this section, we briefly review recent studies that suggest that the radical pair mechanism may explain isotope effects in xenon-induced anaesthesia, and lithium effects on hyperactivity, magnetic field and lithium effects on the circadian clock, and hypomagnetic field effects on neurogenesis and microtubule reorganization.

### 4.1. Xenon anaesthesia

Xenon is a well-known general anaesthetic used for several species, including *Drosophila*, mice and humans [478]. Despite its simple structure (a single atom), the exact underlying mechanism by which it exerts its anaesthetic effects remains unclear. Turin *et al.* showed that when xenon acts anaesthetically on *Drosophila*, specific electron spin resonance (ESR) signals can be observed [479]. The same authors proposed that the anaesthetic action of xenon may involve some form of electron transfer. Moreover, Li *et al.* showed experimentally that isotopes of xenon with non-zero nuclear spin had reduced anaesthetic potency in mice compared with isotopes with no nuclear spin [384]. These findings are consistent with the hypothesis of radical pair creation in xenon-induced anaesthesia.

**Figure 4.**
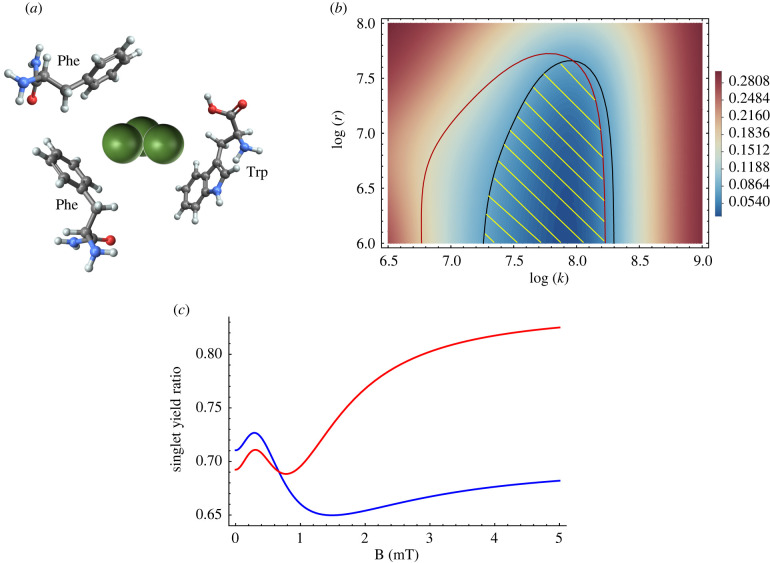
Radical pair explanation for isotope effects in xenon-induced anaesthesia. (*a*) Schematic presentation of the interaction of xenon (green spheres) with aromatic rings of tryptophan (Trp) and phenylalanine (Phe) at the glycine-binding site of the NMDAR [483]. (*b*) The dependence of the agreement between relative anaesthetic potency and singlet yield ratio on the relationship between relaxation rate, *r*, and reaction rate, *k*. The radical pair model can explain the experimentally derived relative anaesthetic potency of xenon, shaded in yellow. (*c*) Predicted dependence of the anaesthetic potency as given by the singlet yield ratio, based on the radical pair model of ^129^Xe/^130^Xe (blue) and ^131^Xe/^130^Xe (red) on an external magnetic field [57].

Franks and co-workers identified the NMDA subtype of glutamate receptor [480] as a target for xenon anaesthesia [478,481]. They further showed that xenon exerted its effects by inhibiting NMDARs by competing with the co-agonist glycine at the glycine-binding site on the GluN1 subunit [482]. Subsequently, the same group identified that xenon interacts with a small number of amino acids at the predicted binding site of the NMDAR [483]. Using grand canonical Monte Carlo method, they showed that xenon at the binding site can interact with tryptophan and phenylalanine, as shown in figure 4*a*. However, due to redox inactivity, it is highly unlikely that phenylalanine can be involved in the electron transfer process [484,485]. Meanwhile, tryptophan is redox active and hence can feasibly be involved in electron transfer and hence the formation of radical pairs, as seen in the context of cryptochrome magnetoreception [43]. In addition, it is known that tryptophan residues of the NMDAR play key roles in channel gating [486,487]. Moreover, exposure to low-intensity magnetic fields activates the NMDAR [228,245,271].

It is also known that ROS are implicated in the activation of the NMDARs [482,488–492]. Moreover, Turin and Skoulakis [493] reported that oxygen gas was necessary for observing spin changes during xenon-induced anaesthesia in *Drosophila*. Motivated by these observations, the authors [57] suggested that the electron transfer related to xenon’s anaesthetic action that is evidenced by Turin *et al.* [479] plays a role in the recombination dynamics of a naturally occurring $\left[{\mathrm{Trp}}^{.+}\cdots O_{2}^{.-}\right]$ radical pair (see §3.4 for further discussion). Using equations (3.19) and (3.20), they showed that for isotopes of xenon with a non-zero nuclear spin, this nuclear spin couples with (at least one of) the electron spins of such a radical pair, affecting the reaction yields of the radical pair and hence xenon’s anaesthetic action. The radical pair was assumed to start off from a singlet state. Such a mechanism is consistent with the experimental results of Li *et al.* [384] that xenon isotopes with non-zero nuclear spin have reduced anaesthetic potency compared to isotopes with zero nuclear spin, as shown in figure 4*b*. The authors also provide an experimental test for the validity of their model (figure 4*c*). It predicts that under a static magnetic field the anaesthetic potency of xenon may be significantly different than that observed by Li *et al.* [384], as shown in figure 4*c*.

### 4.2. Lithium effects on hyperactivity

Lithium (Li) is the most well-known treatment for bipolar illness [494–499]. Despite its frequent clinical use, the mechanism by which Li exerts its effects remains elusive [500]. Ettenberg and co-workers [383] showed that Li effects on the manic phase in rats are isotope-dependent. They used sub-anaesthetic doses of ketamine to induce hyperactivity which was then treated with lithium. They observed that ^6^Li produced a longer suppression of mania compared to ^7^Li. Further, there is a considerable amount of evidence that oxidative stress [130] is implicated in both bipolar disorder [501–509] and its Li treatment [510–513].

**Figure 5.**
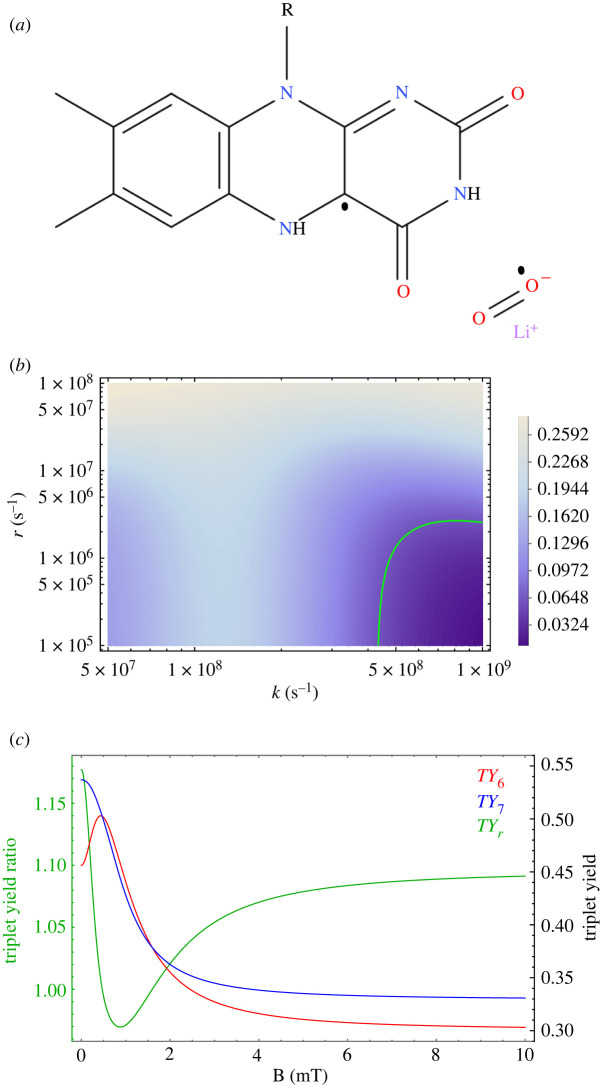
Radical pair explanation for isotope effects in lithium treatment for hyperactivity. (*a*) Flavinsemiquinone (FADH^.^) and lithium superoxide radical pair $\left({\mathrm{Li}}^{+}\cdots O_{2}^{.-}\right)$. (*b*) The dependence of the agreement between the total travelled distance ratio, *TD*_*r*_, and the triplet yield ratio, *TY*_*r*_ of ^7^Li over ^6^Li on the radical pair reaction rate, *k*, and the radical pair spin-coherence relaxation rate, *r*. The green line indicates the ranges smaller than the experimental uncertainty. (*c*) The dependence of the triplet yield (red, ^6^Li; blue, ^7^Li) and triplet yield ratio ^7^Li/^6^Li (green) on an external magnetic field, calculated based on the radical pair model [58].

Bipolar disorder is also correlated with irregularities in circadian rhythms [514–517]. In addition, it is well known that Li influences the circadian rhythms that are disrupted in patients with bipolar disorders [518–531]. Further, Osland *et al.* reported that Li significantly enhanced the expression of *Per2* and *Cry1*, while *Per3*, *Cry2*, *Bmal1*, *E4BP4* and *Rev-Erb-α* expression was decreased [532]. However, the exact mechanisms and pathways behind this therapy are incompletely known. It has been shown that Li can exert it effects via a direct action on the suprachiasmatic nucleus (SCN), a circadian pacemaker in the brain [533–536]. Cryptochromes are key proteins for the circadian clock [537] and SCN’s intercellular networks development, which subserves coherent rhythm expression [538]. Furthermore, it is also shown that cryptochrome is associated with bipolar disorder disease [539–542]. In the context of animal magnetoreception, cryptochromes are the canonical magnetic sensing proteins (See §3.4) [43], with flavin radicals playing a key role. Moreover, it has been shown that circadian rhythms are susceptible to magnetic fields at low intensities [115–117,169–171,224,226,353], where cryptochromes [80,225] are implicated. It has also been observed that cryptochromes play key roles in alteration of ROS levels through exposure to magnetic fields [76,141,222,543]. Based on these facts, a new study suggests [59] that Li’s nuclear spin influences the recombination dynamics of S–T interconversion in the naturally occurring $\left[{\mathrm{FADH}}^{.}\cdots O_{2}^{.-}\right]$ radical pairs (figure 5*a*). These pairs are initially in singlet states, and due to the different nuclear spins, each isotope of Li alters these dynamics differently. Using equations (3.19) and (3.20), the authors showed that a radical pair model could provide results consistent with the experimental finding of Ettenberg and colleagues [383], as shown in figure 5*b*. In that work, it was assumed that the fractional triplet yield of the radical pairs is correlated with lithium potency. They further predict a magnetic field dependence of the effectiveness of lithium, which provides one potential experimental test of their hypothesis, as shown in figure 5*c*.

Furthermore, the authors suggested that the proposed mechanism for Li effects is also plausible via different pathways. Li may exert its effect via competing with magnesium in inhibiting glycogen synthase kinase-3 (GSK-3) [544–546], which is regulated by phosphorylation of inhibitory serine residues [547–549]. GSK-3 phosphorylates the clock components including PER2, CRY1, CLOCK, BMAL1 and REV-ERB*α* [550–557]. In such cases, the radical pairs could be formed in a [RO^.^ · · · Li(H_2_O)n^.^] complex (see §3.4), where RO^.^ is the protein oxy-anion, similar to [65,385,558,559].

### 4.3. Magnetic field and lithium effects on the circadian clock

The circadian clock is essential for the regulation of a variety of physiological and behavioural processes in nearly all organisms, including *Neurospora* [560], *Arabidopsis* [561], *Drosophila* [562], mouse [563] and humans [564–566]. It is known that the disruption of the circadian clock can be detrimental for many physiological functions, including depression [567,568], metabolic and cardiovascular diseases [569], and cancer [570,571]. It is also known that the circadian clock controls physiological processes such as brain metabolism, ROS homeostasis, hormone secretion, autophagy and stem cell proliferation, which are correlated with ageing, memory formation, and neurodegenerative and sleep disorders [572–576]. In *Drosophila*, the circadian clock regulates the timing of eclosion, courtship, rest, activity and feeding; it also influences daytime colour [577] and temperature preference [578]. Regardless of the differences in the molecular components of the circadian clocks, their organization, features, and the molecular mechanism that give rise to rhythmicity are very alike across organisms [579].

Environmental zeitgebers such as light, food and temperature can influence the circadian clock’s rhythmicity [580]. The circadian clock is also susceptible to magnetic field exposures [23,74,171,224–226,353,581,582] (see also §2.1.3). Yoshii *et al.* reported the effects of static magnetic fields with different intensities, [0, 150, 300, 500] μT, on the period changes of *Drosophila*’s circadian clock under blue light illumination [583]. They showed that the period was altered significantly depending on the strength of the magnetic field, with a maximum change at 300 μT. In that work, the geomagnetic field was shielded, and arrhythmic flies were excluded from the analysis. As discussed in §4.2, the disruption of the circadian clock is associated with bipolar disorders, for which Li is the first-line treatment. Li’s effects on bipolar disorder are isotope-dependent. Dokucu *et al.* [584] reported that Li lengthened the period of *Drosophila*’s circadian clock. However, the exact mechanism behind these phenomena is still mostly unknown. Further, ROS homeostasis is correlated to the circadian rhythms [585–591].

**Figure 6.**
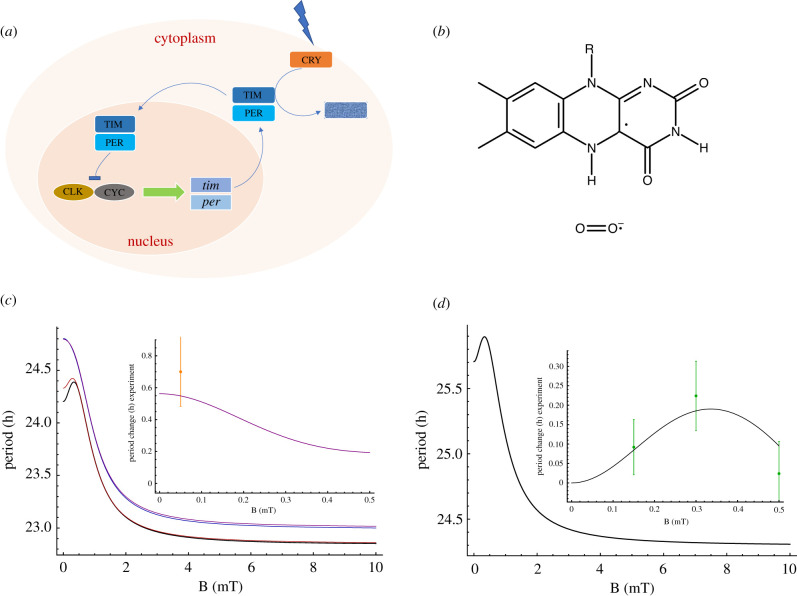
Radical pair explanation for magnetic field and lithium effects on the circadian clock. (*a*) A simple model of the circadian clock feedback loop in *Drosophila*. CLOCK (CLK) and CYCLE (CYC) proteins promote the *tim* and *per* genes. PER and TIM proteins first accumulate in the cytoplasm and then enter into the nucleus to block their gene transcription. Upon light absorption CRY binds to TIM and this results in the degradation of TIM [59]. (*b*) Flavinsemiquinone (FADH^.^) and superoxide radical pair $\left({\mathrm{Li}}^{+}\cdots O_{2}^{.-}\right)$. The dependence of the period of *Drosophila*’s circadian clock calculated by the radical pair model on the static magnetic field strength *B* with (*c*) and without (*d*) lithium effects. Higher magnetic field intensities shorten the period of the circadian clock. (*c*) The effects of Li [purple], ^6^Li [red], ^7^Li [blue] and zero Li [black]. The inset indicates the comparison between the effects of Li on the period of the clock calculated by the radical pair model [purple line] and the experimental findings [orange dots with error bars] of [584]. (*d*) The comparison between the dependence of the period on the applied magnetic field calculated by the radical pair model [black line in the inset of plot (*d*)] and the experimental findings [green dots with error-bars] of [583]. The results from the radical pair model fit the experimental data within the experimental uncertainty.

A recent study suggests that a radical pair model based on $\left[{\mathrm{FADH}}^{.}\cdots O_{2}^{.-}\right]$ (figure 6*b*), similar to §4.2, may explain the magnetic field and lithium effects on *Drosophila*’s circadian clock [59]. Following the work of Tyson *et al.* [592], the authors used a simple mathematical model for *Drosophila*’s circadian clock, as shown in figure 6*a* (for more detailed models see [593]). Similar to the work of Player *et al.* [594], they introduced the effects of applied magnetic fields and hyperfine interactions on the circadian clock process by modifying the corresponding rate representing the role of cryptochrome’s light activation and, hence, proteolysis of protein. Based on these models and using equations (3.19) and (3.20), they reproduced the experimental findings of the magnetic field [583] and lithium effects [584] on *Drosophila*’s circadian clock, as shown in figure 6*c*,*d*. The proposed model in that work predicts that lithium influences the clock in an isotope-dependent manner and magnetic fields and hyperfine interactions modulate oxidative stress in the circadian clock.

### 4.4. Hypomagnetic field effects on microtubule reorganization

Single-cell organisms perform cognitive activities predominantly by cytoskeletal microtubules and are inhibited by anaesthetic gases even in the absence of synapses or networks [595]. Linganna and colleagues showed that modulation of microtubule stability is a mechanism of action for these anaesthetics [596]. Bernard reported that anaesthetics act directly on cytoplasm, depending on cytoskeletal proteins’ dynamics comprising actin filaments and microtubules [597]. Further, Eckenhoff and co-workers found that anaesthetics bind to actin and tubulin [598,599]. In another study, they show that microtubules play key roles in the action of anaesthetics on protein reaction networks involved in neuronal growth, proliferation, division and communication [600]. Despite the low binding affinity of anaesthetics to tubulin compared to membrane protein, the abundance of tubulin is much more than membrane protein sites. It thus seems plausible that our conscious state of mind is intertwined with microtubules and their dynamics.

In recent decades, it has been proposed that quantum physics may explain the mystery of consciousness. In particular, the holistic character of quantum entanglement might shed more light on the binding problem [601]. Penrose & Hameroff proposed that quantum computations in microtubules may be the basis for consciousness [602–604]. It was suggested that electron resonance transfer among tryptophan residues in tubulin in a quantum electronic process could play a role in consciousness [605]. Computational models show that anaesthetic molecules might bind in the same regions and hence result in loss of consciousness [606]. In a recent work, Zhang and co-workers observed a connection between electronic states and vibrational states in tubulin and microtubules [607]. However, quantum electronic coherence beyond ultrafast timescales has been recently challenged experimentally [30]. In contrast, the coherence of quantum spins can be preserved for much longer timescales [608]. Similarly, Fisher has proposed that phosphorus nuclear spins could be entangled in networks of Posner molecules which could form the basis of a quantum mechanism for neural processing in the brain [609]; however, this sort of spin-based model also demands more supporting evidence [610].

A considerable amount of evidence indicates that magnetic fields can influence microtubules [88,611–617]. Wang and colleagues showed that shielding the geomagnetic field caused tubulin assembly disorder [173]. All these observations point to the magnetosensitivity of microtubules for wide ranges of magnetic field strengths. Further, studies suggest that oxidative stress plays important roles in regulating actin and microtubule dynamics [618]. Microtubules contain tryptophan, Tyr and phenylalanine residues which are susceptible to oxidation. Further, it is also known that the stability of polymerized microtubules is susceptible to changes in zinc ion concentration in neurons [619].

**Figure 7.**
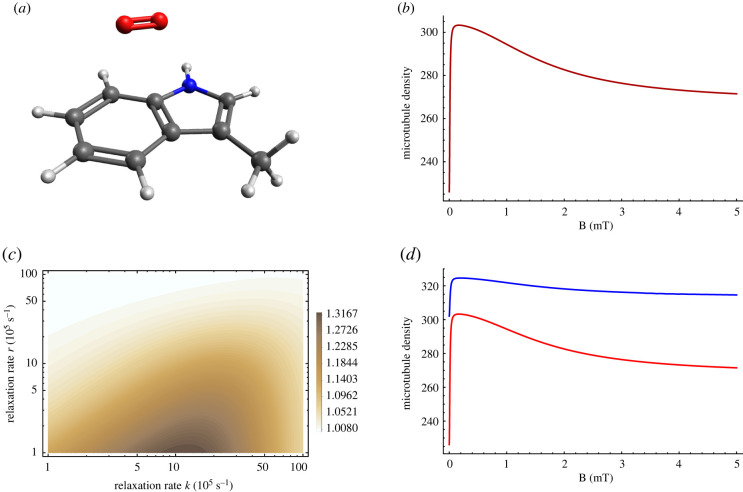
Radical pair explanation for hypomagnetic field effects on microtubule organization. (*a*) Schematic presentation of tryptophan ring and superoxide radicals. (*b*) The dependence of microtubule density on the applied static magnetic field according to a radical pair model based on $\left[{\mathrm{TrpH}}^{.+}\cdots O_{2}^{.-}\right]$ complex. The hypomagnetic field causes a strong decrease in microtubule density. The maximum microtubule density occurs around the geomagnetic field. (*c*) The radical pair model prediction of the microtubule density ratio in the geomagnetic field compared to hypomagnetic field. (*d*) The predicted dependence of microtubule density on administration of Zn (with zero nuclear spin) [red] and ^67^Zn (with nuclear spin of $I_{B}=-\frac{5}{2}$) [blue] as a function of applied magnetic field based on the RP complex of $\left[{\mathrm{TrpH}}^{.+}\cdots O_{2}^{.-}\right]$ [60].

Magnetosensitivty of chemical reactions often involve radical molecules [46]. (See also §3.1.) Using equations (3.19) and (3.20) and a simple kinetic model [619] for dynamics of microtubules, a recent study [60] suggests that a radical pair model in the form of $\left[{\mathrm{Trp}}^{.+}\cdots O_{2}^{.-}\right]$, similar to [57] (see §4.1), may explain the hypomagnetic field effects on microtubule reorganization reported in [173]. They further predict that the effect of zinc on the microtubule density exhibits isotopic dependence, as shown in figure 7.

### 4.5. Hypomagnetic field effects on neurogenesis

In a recent work, Zhang and co-workers showed that shielding the geomagnetic field for a long period (several weeks) decreased neurogenesis in the hippocampal region in mice [172]. They observed that the neurogenesis impairment was through decreasing adult neuronal stem cell proliferation, altering cell lineages in critical development stages of neurogenesis, impeding dendritic development of newborn neurons in the adult hippocampus, and resulting in impaired cognition. Using transcriptome analysis and endogenous ROS *in situ* labelling via hydroethidine, they reported that the hypomagnetic fields reduced levels of ROS [130]. The authors further revealed that such a reduction in reactive oxygen species can be compensated by pharmacological inhibition of ROS removal via diethyldithiocarbamate, which rescued defective adult hippocampal neurogenesis in hypomagnetic field-exposed mice.

Moreover, it is known that the cellular production of ROS is susceptible to magnetic field exposure [136,227,620–637]. ROS play vital roles in biology. The mitochondrial ETC and an enzyme family termed NADPH oxidase are two main cellular sources of ROS [130]. The latter is a flavin-containing enzyme. NADPH oxidase enzymes transport electrons from NADPH, through flavin adenine dinucleotide, across the plasma membrane to O_2_ to produce O_2_^−^ [638].

**Figure 8.**
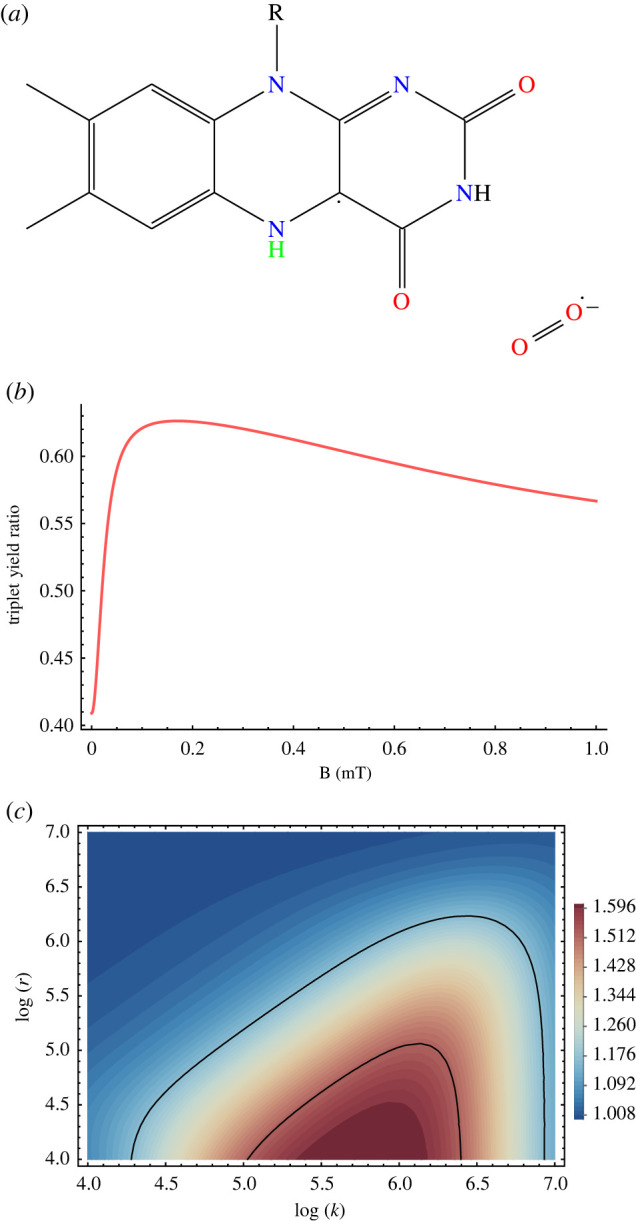
Radical pair explanation for hypomagnetic field effects on hippocampal neurogenesis. (*a*) $\left[{\mathrm{FADH}}^{.}\cdots O_{2}^{.-}\right]$ radical pair. (*b*) The dependence of the triplet yield of the radical pair model for singlet-born radical pair on external magnetic field [61]. (*c*) Triplet yield ratio (geomagnetic field to hypomagnetic field) for singlet-born radical pair in the plan of reaction rate (*k*) and relaxation rate (*r*). The region between the solid black lines is in agreement with the experimental range for the ratio of the numbers of BrdU+ cells after eight weeks, observed in [172].

Based on these findings, a recent study [61] suggests that a radical pair model may explain the modulation of ROS production and the attenuation of adult hippocampal neurogenesis in a hypomagnetic field, observed by Zhang and colleagues [172]. The authors proposed that the reduction of the geomagnetic field influences the spin dynamics of the naturally occurring radical pairs in the form of $\left[{\mathrm{FADH}}^{.}\cdots O_{2}^{.-}\right]$, similar to other studies [58,59,446] (see also §§4.2 and 4.3). They further predict the effects of applied magnetic fields and oxygen isotopic substitution on hippocampal neurogenesis (figure 8)

## 5. Conclusion and outlook

The effects of weak magnetic fields in biology are abundant, including in plants, fungi, animals and humans. The corresponding energies for such effects are far below thermal energies. So far, there is no explanation for such phenomena. However, quantum biology provides a promising explanation for these effects, namely the radical pair mechanism. Here, we have reviewed numerous studies on the biological effects of weak magnetic fields (static and oscillating), as well as related isotope effects. We then reviewed the radical pair mechanism and proposed that it can provide a unified model for weak magnetic field and isotope effects on biology. We discussed candidate radical pairs that may be formed in biological environments. We reviewed recent studies that propose that the radical pair mechanism may explain xenon-induced anaesthesia, lithium effects on mania, magnetic field and lithium effects on the circadian clock, and hypomagnetic field effects on neurogenesis and microtubule reorganization. These recent studies provide avenues for testing the proposed models. For instance, it is proposed that, in xenon anaesthesia, applying magnetic fields over 1mT will increase the anaesthetic potency difference between ^129^Xe and ^131^Xe [57]. Similarly, it is predicted that for mania treatment by ^6^Li and ^7^Li [58] exposure to hypomagnetic and magnetic fields greater than 3 mT will magnify the difference in the potency of these two isotopes. Moreover, it is predicted that exposure of the circadian clock to magnetic fields >mT will shorten the period of the clock [59]. Another study suggests that exposure to magnetic fields greater than the geomagnetic field will reduce microtubule assembly [60]. Further, it is also predicted that hippocampal neurogenesis [61], the circadian clock [59] and microtubule reorganization [60] will be isotope-dependent using different isotopes of oxygen, lithium and zinc, respectively.

It should be noted that the radical pair models used in the studies that we reviewed in §4 are simplified, partly because the exact radical pair molecules involved in these systems are still unknown [117]. This is the case even in the context of avian magnetoreception, where the proposed radical pairs include flavin–tryptophan, flavin–tyrosin and flavin–superoxide among others [43,433]. More realistic models of the radical pairs may provide further insight into the underlying mechanism behind these phenomena. This might involve including multiple nuclei, dipolar, and exchange interactions in the models. It should also be pointed out that including these interactions can reduce the predicted effect size [61,440]. However, this may be balanced by potential amplification effects in the biological systems [59,594].

It has been pointed out that due to fast molecular rotation, free superoxide has a short spin relaxation lifetime on the order of 1 ns, which means a high spin relaxation rate *r* [440,446], which is consistent with the scarcity of observations of superoxide radicals by ESR spectroscopy. The required relaxation rates in the discussed projects in §4 are significantly lower than this expected value. However, it has also been argued that the spin relaxation of free superoxide can be reduced if the molecular symmetry is lowered and the angular momentum is quenched by the biological environment [440,446]. Such conditions might occur if the superoxide molecule is tightly bound [446]. It has also been suggested that the involvement of scavenger species around superoxide can reduce its spin relaxation rate [404–406]. These suggested mechanisms are more complex than the simple radical pair mechanism discussed in this review.

Going beyond these already published proposals, it would be of interest to investigate the roles of radical pairs to help explain magnetic field effects on a large variety of physiological functions, including NMDAR activation [228,245], DNA/RNA methylation [174], dopamine dynamics [251,252], flavin autofluorescence [142], epigenetics [260,261] and many others. As discussed earlier in this review, for each of these systems, there are naturally occurring radical pairs that can conceivably act as magnetosensitive agents. However, in all of the mentioned systems, it remains a major open challenge to definitively identify the magnetic sensitive radical pairs as well as the relevant chemical reactions and corresponding kinetic rates. This challenge will require multi-disciplinary collaborations including biologists, chemists and quantum physicists.

It should be noted that reproducibility of weak magnetic field effects in biology has been a challenge [3,31,408,639–641]. There are several studies reporting failed attempts at independent replications of magnetic field effects in biological systems [5,642–646]. However, this problem is not confined to this particular area of the life sciences. For example, a recent analysis of high-impact cancer studies concluded that only five out of 53 papers could be fully reproduced [647]. A lot of these issues are likely due to the complexity of biological systems [648]. Despite these challenges, it seems unlikely that all of the hundreds of magnetic field effects on biological systems that have been reported are erroneous. One of our main goals in writing the present review was to make the scientific community aware of how many of such studies there are, and how far they go beyond the specific and much more well-known context of avian magnetoreception.

Low level (graeter than 10 nT) radio frequencies from ambient anthropogenic sources present in and around laboratory settings have been observed to influence magnetic compass responses in animals as different a song-birds, murine rodents and amphipods [451,649,650]. Further, it is shown that changes in radio frequencies exposure, not just the presence or absence of an RF field, can alter responses to the static field [650,651]. This may also contribute to the reproducibility issues of magnetosensitivity in biology.

A considerable amount of evidence indicates that shielding the geomagnetic field has direct biological consequences, which in some cases could be detrimental. This could also be pertinent for the quest of life on other planets without a magnetic field, including Mars [652,653]. In a similar vein, nowadays almost all species are exposed to magnetic fields at different intensities and frequencies originated by manufactured devices [70,354,654–658]. The effects of magnetic fields on physiological functions are inevitable and could be detrimental. Thus this review and perspective is pertinent to the debate on the putative adverse health effects of environmental magnetic fields. Understanding the underlying mechanism should help to clarify many of these issues.

It would be of interest to further investigate the role of cryptochrome proteins in magnetic sensitivity in biology. However, it is equally important to search for candidate molecules other than cryptochromes that could be involved in magnetosensitivity involving a radical pair mechanism.

It is also of interest to explore other potential mechanisms for magnetosensitivity beyond the radical pair mechanism, such as magnetites. The high sensitivity necessary to detect spatial variation in the inclination (approx. 0.01° km^−1^) or intensity (3–5 nT km^−1^) may be relevant to the effects that are discussed in this review [41]. It is well established that migratory birds and sea turtles use a magnetic map for navigation. However, a recent study suggests that a short-range, high-resolution map may be used by vertebrates that move only a few kilometres (newts, deer mice) [659]; this may help explain claims over the years that temporal fluctuations in the magnetic field could provide a zeitgeber for the entrainment of circadian rhythms. The link between high sensitivity responses to the magnetic field and circadian rhythmicity might be relevant to some of the ‘non-specific’ effects discussed in this review. Another interesting avenue for magnetosensitivity is the involvement of scavenger species in the radical pair mechanism, which leads to radical triads [404–406,660].

From a quantum perspective, it would also be of interest to explore the relevance of quantum entanglement [661] in the radical pair models for various magnetic field effects on biological functions [662–664]. This could be particularly interesting in the context of neuroscience, where it has been suggested that the brain might use quantum effects such as entanglement for information processing purposes [605,609,665].

Studying magnetic field and isotope effects in biology is a rich and important interdisciplinary field. The potential essential involvement of quantum effects related to the radical pair mechanism provides an exciting new avenue for further investigation, with the promise of revealing a common underlying mechanism for many of these effects.

## Data Availability

This article has no additional data.
